# Conformational heterogeneity of the calmodulin binding interface

**DOI:** 10.1038/ncomms10910

**Published:** 2016-04-04

**Authors:** Diwakar Shukla, Ariana Peck, Vijay S. Pande

**Affiliations:** 1 Department of Chemistry, Stanford University, Stanford, California 94305, USA; 2 SIMBIOS NIH Center for Biomedical Computation, Stanford University, Stanford, California 94305, USA; 3 Department of Chemical and Biomolecular Engineering, University of Illinois at Urbana-Champaign, Urbana, Illinois 61801, USA; 4 Department of Biochemistry, Stanford University School of Medicine, Stanford, California 94305, USA

## Abstract

Calmodulin (CaM) is a ubiquitous Ca^2+^ sensor and a crucial signalling hub in many pathways aberrantly activated in disease. However, the mechanistic basis of its ability to bind diverse signalling molecules including G-protein-coupled receptors, ion channels and kinases remains poorly understood. Here we harness the high resolution of molecular dynamics simulations and the analytical power of Markov state models to dissect the molecular underpinnings of CaM binding diversity. Our computational model indicates that in the absence of Ca^2+^, sub-states in the folded ensemble of CaM's C-terminal domain present chemically and sterically distinct topologies that may facilitate conformational selection. Furthermore, we find that local unfolding is off-pathway for the exchange process relevant for peptide binding, in contrast to prior hypotheses that unfolding might account for binding diversity. Finally, our model predicts a novel binding interface that is well-populated in the Ca^2+^-bound regime and, thus, a candidate for pharmacological intervention.

## Abstract (web summary)

Calmodulin is an important calcium sensor that can recognise and interact with a large range of proteins. Here, the authors use molecular dynamics simulations and Markov state models to show that the range of binding conformations adopted without substrate can help explain its diverse and specific binding.

## Graphical abstract

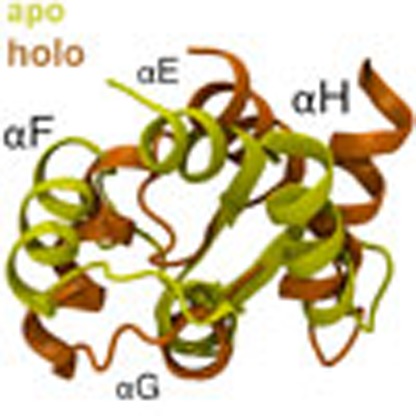

Calcium signalling plays a pivotal role in diverse physiological processes, including lymphocyte activation, neuronal firing, muscle contraction and apoptosis^1^. Under resting conditions, cells maintain a 20,000-fold gradient in Ca^2+^ concentration across the plasma membrane that is rapidly dissipated and restored after stimulation^2^. As a consequence, hundreds of Ca^2+^-binding proteins have evolved whose affinity for Ca^2+^ collectively spans the nM to mM range^2^,^3^. Broadly, these can be classified into proteins that act as Ca^2+^ sinks to buffer the intracellular environment and adaptor proteins that act as Ca^2+^ sensors, undergoing large conformational changes upon binding to carry out distinct effector functions in the absence and presence of Ca^2+^ (ref. 4).

Calmodulin (CaM) is the prototypical eukaryotic Ca^2+^ sensor and regulates a diverse set of proteins, including G-protein-coupled receptors, small conductance potassium (SK) channels and CaM kinases^4^,^5^. Structurally, CaM is comprised of two lobes connected by a short linker^6^. Each lobe, in turn, contains a pair of EF hand motifs that bind two Ca^2+^ ions cooperatively^7^. Extensive NMR and small-angle X-ray scattering studies have shown that CaM is a dynamic protein, with its inter-domain linker exhibiting considerable flexibility and the C-lobe undergoing conformational exchange in the μs regime^8^,^9^. Comparison of high-resolution structures of apo (Ca^2+^-unbound) and holo (Ca^2+^-bound) CaM clarified the functional role of these dynamics by indicating that Ca^2+^ binding induces large-scale structural rearrangements that expose each lobe's hydrophobic interface to CaM-binding partners^6^,^10^ (Fig. 1). These binding partners commonly feature a pair of bulky hydrophobic residues that serve as anchors to the hydrophobic interface presented by each lobe of Ca^2+^-bound CaM, which can accommodate variable spacing between these anchor residues due to the flexibility of the inter-lobe linker^11^.

However, this prevailing paradigm of CaM function has been challenged by the discovery of proteins that preferentially bind to apo CaM and tune its affinity for Ca^2+^ (refs 5, 12). Furthermore, CaM-binding partners share no sequence homology^4^, and attempts to categorize them based on structural features are riddled with exceptions^11^,^13^. Thus, a holistic framework that accounts for CaM's binding diversity is lacking. Prior computational work has focused on CaM equilibrium dynamics, with particular emphasis on dissecting the temperature- and Ca^2+^-dependence of the transition rates among the unfolded, apo- and holo-states and their relative populations^14^,^15^. While these studies have helped resolve conflicting interpretations of NMR and single-molecule force spectroscopy studies by distinguishing between states that are difficult to resolve experimentally^14^,^16^,^17^, they have been too coarse-grained to address the mechanism of these transitions at atomistic detail. Furthermore, only the question of Ca^2+^-binding has been addressed, while the structural basis underlying CaM's ability to interact specifically with a diverse set of binding partners has not been broached computationally.

Molecular dynamics (MD) simulations have been harnessed to study the dynamics of complex biological systems at atomic resolution and predict the mechanisms that underlie transitions among biologically important states^18^,^19^,^20^. Two challenges faced by MD simulations are the need for a framework to deal with the high dimensionality of the resulting data set and the requirement of highly specialized hardware to obtain atomistic dynamics of proteins with timescales comparable to the conformational process under investigation^21^. Markov state models (MSMs) are a powerful analytical tool that overcomes both obstacles by merging massively parallel short simulations into a unified, more accessible model that captures rare events whose timescales exceed those of the individual simulations^21^,^22^.

In this study, we constructed MSMs from MD simulations of the C-lobe of CaM (C-CaM) in the Ca^2+^-bound and unbound regimes to explore the contributions of a single domain of CaM to binding diversity. In particular, we performed MD for a total aggregate simulation time of 700 μs with an all-atom description of protein and an explicit representation of water. This study is in contrast to previous computational investigations of CaM conformational dynamics, which relied either on coarse-grained models^14^ or short (ns) simulations^23^,^24^. Our MSM-based approach resolved the experimentally observed apo to holo and unfolding transitions as orthogonal processes, while comparison with available high-resolution structures indicated that the folded ensemble of C-CaM populates multiple, topologically distinct binding interfaces that may account for binding specificity and diversity by enabling conformational selection. In addition, our models predict novel binding interfaces that could be stabilized by structurally matched targets, thus disrupting downstream signalling by sequestering CaM from the active pool of signalling molecules. Given that CaM is a pivotal hub in signalling pathways that are frequently deregulated in disease^25^,^26^, a sophisticated framework for understanding target recognition by CaM could help make it a tractable candidate for pharmacological activation or inhibition.

## Results

### Distinct exchange processes characterize apo- and holo-C-CaM

**Figure 1:**
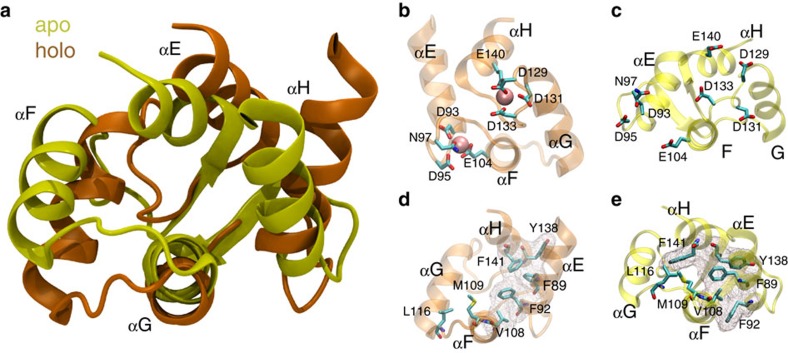
C-CaM conformational change upon Ca^2+^ binding. (**a**) Comparison of the C-terminal domain from published structures of apo (1CFD) and holo (1CLL) CaM indicates the large-scale structural rearrangements that occur when Ca^2+^ binds. (**b**,**c**) In particular, repositioning of the Ca^2+^-ligating residues from solvent-exposed rotamers renders each EF hand competent to bind Ca^2+^. (**d**,**e**) Additionally, binding of Ca^2+^ induces repacking in the hydrophobic core and binding interface, enabling compact clustering of the aromatic residues outlined by the mesh.

To investigate the contributions of C-CaM to CaM's binding diversity, we ran MD simulations of this domain in the absence and presence of Ca^2+^. Inspection of the corresponding high-resolution structures of apo- and holo-CaM from which these simulations were started demonstrates the gross structural rearrangements that occur upon Ca^2+^ binding (Fig. 1a)^27^,^28^. In addition to repositioning of the residues that ligate the Ca^2+^ ions in each of the Ca^2+^-binding sites (Fig. 1b,c), Ca^2+^ binding is accompanied by reorientation of the EF hand α-helices to achieve a more compact clustering of the four aromatic residues in the C-CaM hydrophobic core (Fig. 1d,e). However, these are static depictions that offer atomic-level structural information but minimal insight into the dynamics that govern each state or the mechanism underlying their exchange. MD simulations allowed us to bridge this gap, preserving the atomic-level detail afforded by high-resolution structures while providing extensive kinetic information that can be unified and interpreted through the framework of MSMs.

Constructing MSMs that accurately recapitulate MD data requires a valid decomposition of phase space. We partitioned the conformational landscape explored by apo- and holo-C-CaM using time-structure-based independent component analysis (tICA), a distance metric generated by projecting high-dimensional data onto degrees of freedom (in this case, distances in the contact map) that decorrelate slowly^29^. MSMs were then built from the clustered data at a Markovian lag-time (Supplementary Fig. 1) to analyse the dominant pathways of conformational exchange. Building MSMs from the tIC-partitioned phase space allowed us to analyse the rates and pathways of conformational change that govern apo- and holo-C-CaM dynamics. The first eigenvector of the transition probability matrix defining each MSM provides an estimate of the equilibrium populations. In the absence of bound Ca^2+^, conformations similar to the solution structure of apo-C-CaM dominate, with the holo-like state accounting for a minor but significant population (*P*∼0.14), similar to the minor 5–10% population observed in NMR studies^9^,^27^,^30^. These values are within 1 kcal of each other and thus difficult to distinguish experimentally or computationally (Supplementary Fig. 2a). By contrast, most of the population in the Ca^2+^-bound regime is concentrated in a state that resembles the crystal structure of holo-C-CaM (*P*∼0.93) (Supplementary Fig. 2b). To further validate these computational models, we estimated NMR order parameters from each MSM ensemble and found good agreement with experimental values (Supplementary Fig. 3 and Supplementary Tables 1–2)^30^,^31^,^32^ These order parameters measure local flexibility, so the high correlation suggests that the MSM ensembles accurately reproduce solution-state dynamics. We additionally verified that the conformational landscape explored by apo-C-CaM was invariant to force field choice by re-running our simulations in a different force field. The population distributions along order parameters characteristic of the apo- to holo-like transition and local unfolding were similar between apo-C-CaM simulations run in distinct force fields, and equally distant to the population distribution of the holo system (Supplementary Fig. 4 and Supplementary Table 3).

**Figure 2:**
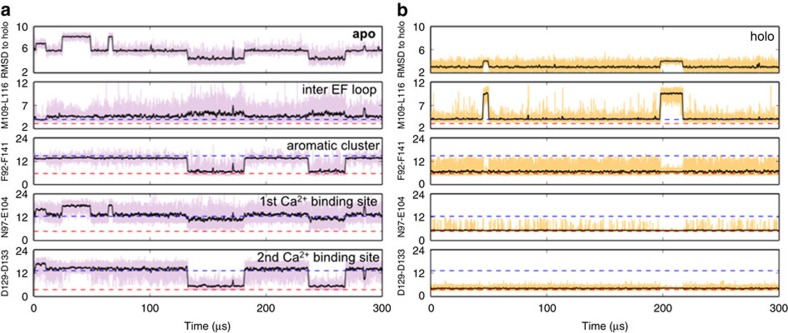
Kinetics of conformational change. Sampling from the MSM captures conformational changes and the timescales at which they occur. (**a**) In apo-C-CaM, transition to a holo-like state coincides with aromatic stacking and formation of the second, but not the first Ca^2+^-binding site. (**b**) Holo-C-CaM experiences fluctuations in the inter-EF hand loop region but does not show sustained fluctuations in the aromatic cluster or either Ca^2+^-binding site. All distances are shown in Å.

Sampling from this equilibrium description recovers the dynamic processes and the timescales at which they occur. In the absence of Ca^2+^, exchange with the holo-like state occurs with a *k*_ex_ of ∼26 μs, within the range of 18–150 μs that has been experimentally determined for the isolated C-terminal domain (Fig. 2a)^30^,^33^,^34^,^35^,^36^,^37^. Transitions to the holo-like state coincide with aromatic stacking and formation of the second Ca^2+^-binding site, as assessed by inter-residue distances between Phe92 and Phe141 and between the Ca^2+^ ligands Asp129 and Asp133, respectively. On the other hand, the first Ca^2+^-binding site remains poorly formed even upon transition to the holo-like state, which likely accounts for its lower affinity for Ca^2+^ (ref. 30) and structurally distinguishes the holo-like and holo states. Exchange along these order parameters correlates well with the second eigenvector (Supplementary Fig. 5a), indicating that the apo- to holo-like transition is the slowest dynamic process in the absence of Ca^2+^.

By contrast, motions in the loop connecting the EF hands account for the slowest dynamic process of the holo system, with a relaxation timescale of ∼10 μs (Fig. 2b). Specifically, twisting of this region reorients Leu116 away from solvent, allowing it to pack against Met124 and Met109, while adjacent Thr117 flips away from the hydrophobic cluster in this region (Supplementary Figs 5b, 6). These dynamics are not characteristic of the apo- to holo-like transition. Similarly, the conformational change processes that distinguish the apo MSM do not typify the excursions from the dominant population in the holo MSM, indicating that that the dynamics in the Ca^2+^-bound and unbound regimes are distinct (Fig. 2).

### Computationally discovered metrics reveal complex dynamics

**Figure 3:**
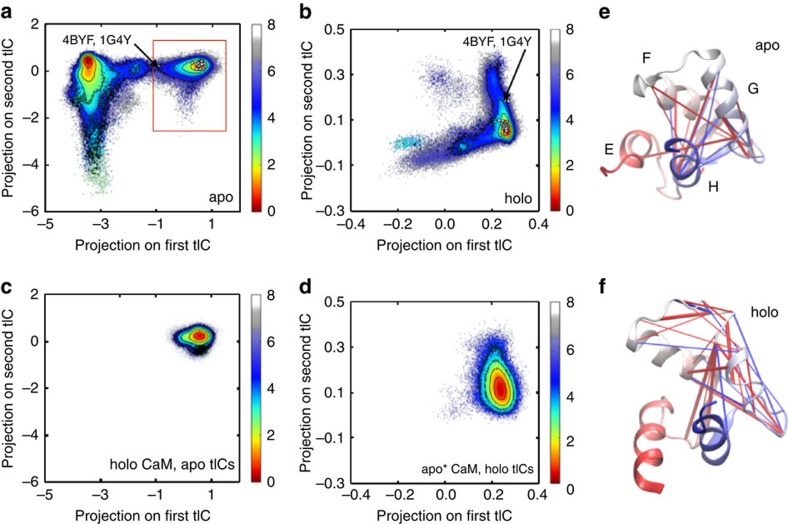
The top tICs capture distinct dynamic processes. The energy landscapes of (**a**) apo- and (**b**) holo-C-CaM were generated by projecting the system's conformations, weighted by their MSM probabilities, onto their respective tICs. Comparison was made to available experimental structures by projecting the C-lobe of these published structures onto the relevant tICs (white dots). (**c**) Projection of the holo conformations on the apo tICs and (**d**) the holo-like basin in the apo system (boxed in red in **a**) on the holo tICs underscores their different dynamics. Free energy values are reported in kcal mol^−1^. The 25 inter-residue distances whose dynamics most contribute to the top (**e**) apo and (**f**) holo tIC are visualized on cartoon representations of the published structures of apo- and holo-C-CaM. Red and blue lines denote positive and negative tIC values, respectively.

The ability of our MSMs to recover experimentally determined populations and rates indicates that tICA is a robust method for discretizing the phase space explored by apo- and holo-C-CaM. The dominant tICs—that is, those with the largest eigenvalues in the time-lag covariance matrix—represent the slowest decorrelating orthogonal linear combinations of distances in the contact map^29^. Thus, deducing the molecular correlates of the tICs provides insight into the most important degrees of freedom in the conformational landscape of each system. Projecting the conformations visited by apo- and holo-C-CaM onto their respective tICs confirmed that within each system, the top tICs capture distinct dynamic processes (Fig. 3a,b). In the apo system, the first and second tICs correspond to exchange with a holo-like form and a partially unfolded state, respectively (Supplementary Fig. 7a), consistent with prior studies that identified these as the dominant dynamics in the unliganded regime^14^,^17^,^30^. Projecting the conformational space explored by holo-C-CaM onto these same tICs resulted in a density map that overlaid well with the holo-like state explored by apo-C-CaM, but there was no indication that holo-C-CaM populates an apo-like state or undergoes the cracking experienced by the Ca^2+^-unbound state (Fig. 3c).

Instead, the dominant tICs that characterize holo-C-CaM correspond to different rearrangements of the hydrophobic core (Supplementary Fig. 7b). However, these tICs are specific to the holo system, and the first tIC in particular fails to capture the dynamics of the holo-like state of apo-C-CaM (Fig. 3d), again underscoring that dynamics of C-CaM in the Ca^2+^-bound and unbound regimes are distinct. Visualizing the dominant tIC of each system as a contact map confirms this: the region that most contributes to the top tIC is localized to the G helix and inter-EF hand loop region for holo-C-CaM but more distributed throughout the protein for apo-C-CaM (Fig. 3e,f and Supplementary Fig. 8). The complexity of these contact maps underscores the need for order parameters that can capture such intricate and non-uniform dynamics. Indeed, the commonly used order parameter of root-mean-square distance (RMSD) provides much poorer separation of the apo- and holo-like basins in the Ca^2+^-unbound regime, and fails to resolve dynamic exchange in the Ca^2+^-bound regime entirely (Supplementary Fig. 9).

### Hydrophobic repack dominates the apo to holo-like transition

**Figure 4:**
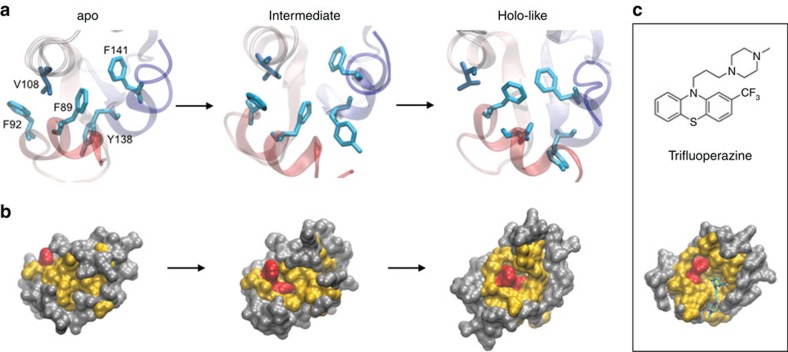
Hydrophobic repack determines the topology of the binding interface. (**a**) Packing of Val108 between Phe92 and Phe89 is disrupted during the apo- to holo-like transition, which enables the formation of a compact aromatic cluster in the C-CaM core. (**b**) Surface representations of the apo, intermediate, and holo-like states show that repack exposes distinct surface topologies. Phe, all other hydrophobic, and polar residues coloured red, yellow and grey, respectively. (**c**) The Phe-lined cavity formed in the holo-like state is commonly accessed by bulky aromatic rings of binding partners, as shown in the crystal structure of holo CaM in complex with trifluoperazine. Only the C-terminal domain of this structure (1CTR) is shown.

Applying transition pathway theory to MSMs predicts the ensemble of pathways between states and estimates their associated fluxes. In the apo MSM, a structural rearrangement characteristic of the transition to the holo-like state is compact stacking of four aromatic residues in the hydrophobic core, which is impeded in the apo state by packing of Val108 between Phe89 and Phe92 (Fig. 1d,e). Over 60% of the flux passes through an intermediate, in which packing of Val108 against Phe89 (but not Phe92) is disrupted, but only in the holo-like state does the helix containing Val108 shift sufficiently to accommodate the compact aromatic cluster (Fig. 4a). Repacking of the core orients Phe89 and Phe141 in the bottom of a hydrophobic cavity, an arrangement that mimics the surface topology of Ca^2+^-bound C-CaM and is late to form along the principle transition paths (Fig. 4b). Interestingly, a common structural motif in CaM's diverse binding partners is the presence of a bulky aromatic feature that accesses this hydrophobic pocket and stacks against the Phe residues that line one side of the cavity (Fig. 4c)^38^,^39^. Thus, rearrangements in the hydrophobic core facilitate the presentation of a specific surface topology that contributes to binding specificity.

**Figure 5:**
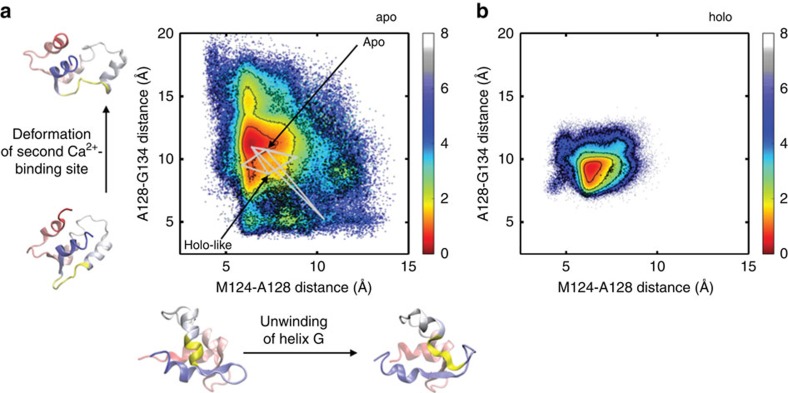
Local unfolding occurs in apo but not holo-C-CaM. The energy landscapes of (**a**) apo and (**b**) holo-C-CaM as a function of the Met124-Ala128 and Ala128-Gly134 inter-Cα distances, which correlate with distortions in helix G and the second Ca^2+^-binding site, respectively. The regions that span these residues are highlighted in yellow in the cartoon representations below and to the side of the plot in (**a**). Conformations were weighted by their MSM probabilities, and free energy values are reported in kcal mol^−1^. In **a**, the five paths with the greatest flux between the apo- and holo-like states are plotted as grey lines.

Another intriguing question about the mechanism underlying this transition is whether unfolding plays a role. An unfolding process on the μs timescale has been observed in T-jump spectroscopic studies^17^, and although exchange with the holo-like state rather than the unfolded state is predicted to dominate at low temperatures, the unfolded population is still estimated to be 1% in this regime^14^. Our MSM of apo-C-CaM populates a landscape that is qualitatively similar to the one explored by the coarse-grained Gö model of Chen *et al*.^14^ (Supplementary Fig. 10). However, the higher resolution of our simulations reveal that unfolding is not global, but rather a localized phenomenon that primarily involves unwinding of helix G or deformation of the second Ca^2+^-binding site (Fig. 5a). Less than 3% of the flux between the apo- and holo-like states visits a partially unfolded intermediate (using lower cutoffs of 11 and 15 Å for the Met124-Ala128 and Ala128-Gly134 inter-C*α* distances, respectively, to categorize states as partially unfolded). This indicates that unfolding is largely irrelevant for the apo- to holo-like transition, despite prior speculation that intrinsic disorder within C-CaM might be a mechanism underlying its diverse binding repertoire^14^. Furthermore, we find no evidence of partial unfolding in the restricted dynamics of holo-C-CaM (Fig. 5b), consistent with the observation of increased stability in the Ca^2+^-bound regime^40^. Because force field choice has been shown to impact secondary structure stability, we compared simulations run in different force fields to ensure that the observed local unfolding was invariant to this choice. As has been previously observed^41^, median helical content is slightly higher in the CHARMM36 simulations than the Amber99sb-ildn trajectories (Supplementary Fig. 4c). However, these distributions are broad and overlap between the different force fields. Furthermore, the conclusions drawn about local unfolding are made based on specific inter-residue distances, which show similar distributions between force fields, rather than helical content (Supplementary Fig. 4a,b).

### Apo- and holo-C-CaM populate topologically distinct ensembles

**Figure 6:**
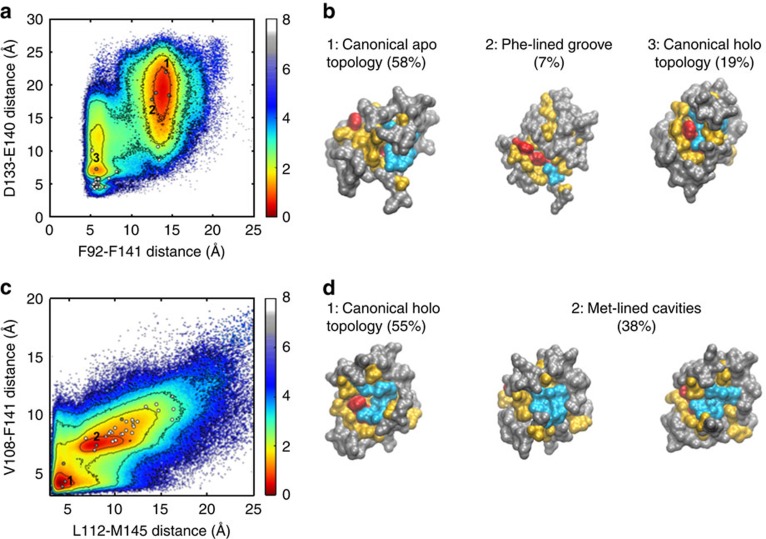
C-CaM presents an ensemble of hydrophobic interfaces in the absence of binding partners. (**a**) Energy landscape of apo-C-CaM as a function of the Phe92-Phe141 and Asp133-Glu140 inter-residue distances, which correspond to aromatic clustering and formation of the second Ca^2+^-binding site, respectively. White and grey dots represent these values for published structures and topological cluster centres, respectively. (**b**) Surface representations of the cluster centres indicated by number in **a**, with Phe, Met, all other hydrophobic, and polar residues coloured red, cyan, yellow and grey, respectively. (**c**,**d**) As in (**a**,**b**), except for holo-C-CaM, with inter-residue distances between hydrophobic residues at the binding interface (specifically, Leu112-Met145 and Val108-Phe141) used as order parameters. Free energy values are reported in kcal mol^−1^.

Given the unimportance of unfolding to the ability of C-CaM to bind structurally dissimilar substrates, we sought to identify a mechanism that could explain this binding diversity by comparing published structures of CaM-target complexes to our MSMs. Mapping 25 of these high-resolution structures to tIC space revealed that C-CaM explores the configurations stabilized by its peptide and small molecule-binding partners even in their absence (Fig. 6). Because our simulations started only from the published apo and holo structures, recovering these other conformations adopted in the presence of CaM-binding partners was not guaranteed. Instead, this pre-existing heterogeneity suggests that conformational selection may facilitate CaM's binding diversity. As expected, most of the structures mapped to the holo-like and central basins in apo- and holo-tIC space, respectively (Fig. 3a,b). The two outliers were the structures of CaM in complex with myosin and an SK channel splice variant (4BYF and 1G4Y, respectively); in both crystal structures, the C-lobes were found to be apo, consistent with their physiological role^42^,^43^.

Curiously, in apo-tIC space these outliers mapped to an intermediary position between the apo- and holo-like basins rather than fully in the former basin despite the absence of Ca^2+^ (Fig. 6a). As described above, transition between these basins involves extensive structural reorganization, including formation of the second Ca^2+^-binding site and hydrophobic repack. Because the first tIC encompasses both rearrangements, we examined the extent to which these features are coupled in the dynamics of apo C-CaM. Mutual information calculations provide a measure of long-range coupling between residues by computing the correlation between the motions of their backbone and side-chain torsion angles^44^. Applying this analysis to apo-C-CaM indicated extensive allosteric communication between the second Ca^2+^-binding site and the hydrophobic residues that constitute the binding interface (Supplementary Fig. 11). In particular, the aromatic residues Phe89 and Tyr138 (Supplementary Fig. 12) show a strong coupling with the charged residues (Asp129, Asp131 and Asp133) that ligate the Ca^2+^ ion. By contrast, the first Ca^2+^-binding site, which has lower affinity for Ca^2+^ (ref. 30), does not show a strong dynamical coupling with any region of the protein (Supplementary Fig. 10).

The majority of flux between the apo- and holo-like states passes through a reaction tube, in which these features change in a concerted fashion, while configurations in which the Ca^2+^-binding sites are fully formed but the aromatic cluster is not (and vice versa) are energetically destabilized by several kT (Supplementary Fig. 13). Consistent with this, splice variants of an SK channel that are known to differentially tune the affinity of the C-lobe for Ca^2+^ stabilize distinct hydrophobic surface topologies. While the splice variant that enhances Ca^2+^ binding interacts with the canonical Phe-lined cavity, the SK2-a splice variant that abolishes Ca^2+^ binding stabilizes a less compact Phe-lined groove that is also observed in the structure of apo CaM in complex with myosin (Supplementary Fig. 14)^5^,^42^,^43^. An exception to the concerted rearrangements in the hydrophobic network and Ca^2+^-binding sites was observed in the NMR structure of CaM in complex with phosphorylated nitric oxide synthase, which features the canonical holo-pocket topology but has poorly formed Ca^2+^-binding sites (Supplementary Fig. 14). However, in this case it has been suggested that proximity of the phosphate to the second Ca^2+^-binding site may prevent closure of the charged residues that ligate Ca^2+^, thus decoupling the allosteric network by an unusual mechanism^12^.

Despite the sequence dissimilarity between the regions of myosin and SK2-a that bind C-CaM, the Phe-lined grooves they stabilize are strikingly similar (Supplementary Fig. 14)^42^,^43^. To predict which other surface topologies are well-represented and thus amenable to conformational selection, we clustered configurations from equilibrium sampling of the apo and holo MSMs based on RMSD of the 10 residues that constitute the hydrophobic network in C-CaM. In the case of the apo system, cluster centres that resembled the canonical apo and holo topologies were recovered, with similar populations (*P*∼0.58 and 0.19, respectively,) to the apo- and holo-like states in the original MSM. Additionally, a Phe-lined groove similar to the one stabilized by myosin and SK2-a was observed at lower frequency (*P*∼0.07) (Fig. 6a,b and Supplementary Fig. 14).

In the case of the holo system, the canonical Phe-lined cavity is most commonly observed (*P*∼0.56), while a significant alternative topology features a Met-lined pocket (*P*∼0.38) (Fig. 6c,d). Although this latter topology is not observed in the repertoire of currently published CaM structures, we predict that it could be readily stabilized by conformational selection based on its distinctive topology and high population in the Ca^2+^-bound regime. This prediction is bolstered by the experimental observation of a binding interface that, in terms of the relative positioning and exposure of the Phe and Met clusters, is intermediary between the canonical Phe-lined cavity and our predicted Met-lined pocket (Supplementary Fig. 15). In this case, an anti-microtubular agent stabilizes an unusual topology featuring an exposed aromatic cluster adjacent to a Met-bordered depression^45^, supporting the potential for other small molecules to sequester alternative topologies presented by holo-C-CaM. Interestingly, the Phe-lined cavity and Met-lined pocket we observe in our simulations are not only sterically but also chemically distinct. As noted above, the Phe cavity provides the opportunity for aromatic stacking and thus commonly accommodates the indole or benzyl rings of CaM-binding partners^38^,^39^. Similarly, the greater polarizability of Met compared with more canonical hydrophobic residues is expected to endow the Met pocket with distinct binding opportunities.

## Discussion

Despite the wealth of structural information of CaM in complex with a variety of its binding partners, a unified framework that accounts for its ability to interact specifically with a diverse repertoire of proteins and small molecules has been lacking. In this study, we use MSMs to join massively parallel, short MD simulations of the isolated C-lobe in the Ca^2+^-bound and unbound regimes to arrive at a more holistic understanding of the dynamics that give rise to CaM's functional plasticity.

Constructing MSMs requires partitioning phase space with distance metrics that distinguish important degrees of freedom in a protein's dynamic landscape. The complexity of the structural features captured by tICA suggests that even for a single domain of a small protein, simple order parameters are insufficient to describe the intricate dynamics of conformational change. Linear combinations of several simple order parameters have already been used to study conformational change; however, the order parameters in these studies were chosen based on already available structural information^18^,^19^,^46^,^47^. In this study, we show that computationally discovered order parameters can readily distinguish between the conformational space explored by apo- and holo-C-CaM, despite being agnostic to available structural information. Furthermore, the tICA method, which identifies slowly decorrelating degrees of freedom in the contact map, provides a superior separation of dynamic processes that are too subtle or complex for more global metrics like RMSD.

Using this unbiased approach to identify the dominant dynamics of C-CaM, we investigated the origins of CaM's ability to bind structurally diverse protein targets. Recently, intrinsic disorder has gained considerable attention as a mechanism for generating binding diversity: initial contacts formed between an intrinsically disordered protein and different substrates seed the formation of distinct secondary structures^48^. However, we find that the unfolding process of apo-C-CaM previously observed^14^,^17^ is irrelevant to the apo- to holo-like transition and thus off-pathway for target binding. In contrast to the induced fit mechanism of intrinsic disorder, our simulations indicate that conformational sub-states within the folded ensemble of apo-C-CaM present distinct surface topologies that may facilitate binding of diverse targets. Although a direct comparison of target binding to different sub-states of CaM would be required to determine whether induced fit or conformational selection dominates, our simulations indicate that the pre-requisite heterogeneity for the former mechanism exists. Furthermore, the sub-states' relative populations predicted by our models can be used to estimate the fold-increase in binding rate required for one mechanism to outcompete the other. Prior studies investigating the process of peptide binding have primarily focused on dynamics of the holo state^31^,^49^,^50^, but our models indicate that the apo ensemble also presents binding interfaces stabilized in crystal structures of CaM-peptide complexes. Furthermore, allosteric communication between the hydrophobic network and the Ca^2+^-binding sites provides an opportunity for binding of a target protein to tune the affinity of CaM for Ca^2+^ and vice versa^5^,^12^,^51^.

Hydrophobic interactions are commonly viewed as relatively non-specific compared with salt bridges and hydrogen bond networks, but the hydrophobic binding interfaces presented by the apo- and holo/holo-like structures are sterically and chemically distinct and thus can provide specificity. Given the observed conformational selection, we used our MSMs to identify alternative binding interfaces that are well-populated by apo- and holo-C-CaM that we predict could be readily stabilized by a structurally matched substrate. As expected, we recovered the Phe-lined cavity and groove topologies that have been observed in crystal structures of CaM-target complexes at high frequency^5^,^52^,^53^. In addition, we identified a well-populated topology featuring a distinctive Met-lined cavity in the holo MSM that, to our knowledge, is not represented in the current repertoire of published CaM structures.

A necessary consideration in the face of the observed conformational heterogeneity is the choice of force field. The Amber-99sb-ildn force field used in this study has been shown to have complex effects on stability, tending to destabilize helical structures but predicting overly compact non-native conformations on folding pathways because of poor solvation^41^,^54^,^55^. However, these studies also suggest that such systemic errors primarily affect simulations of small peptide systems, while good agreement is found between experiment and the ensembles of larger model systems^41^,^56^. Given that our models likewise reproduce experimental structural information, predict substantial heterogeneity in non-helical regions shown previously to be highly flexible, and are similar between different force fields, we do not expect force field choice to affect the conclusions drawn in this study.

Given the distinct kinetic and thermal properties of the two lobes of CaM^9^,^40^, it will be of interest to assess the extent to which conformational heterogeneity in N-CaM contributes to full CaM's binding diversity. NMR experiments indicate that C-CaM is more dynamic than N-CaM^9^, so it is possible that the ensemble of binding interfaces of the former contributes most to binding diversity, while binding of the latter primarily enhances the affinity of this interaction. Another possibility is that N-CaM features a distinct ensemble of binding interfaces that could, in combination with the ensemble populated by C-CaM, be used as an opportunity to effect functional bifurcation, a phenomenon that has already been reported in CaM's physiological function^57^,^58^. CaM plays a pivotal role in signal transduction pathways that are frequently perturbed in disease states^25^,^26^; therefore, a nuanced understanding of its binding modes could facilitate the rational targeting of this signalling hub for pharmacological intervention.

## Methods

### Simulation details for apo CaM

The starting structures were taken from the crystal structure of apo CaM (PDB: 1CFD (ref. 27)). The all-atom structures were then solvated in a cubic box with box length of ∼65 Å with TIP3P (ref. 59) water molecules such that water extended at least 10 Å away from the surface of the protein; 24 Na^+^ ions and 12 Cl^−^ ions were added to the system to neutralize the charge. Covalent bonds involving hydrogen atoms were constrained with the LINear Constraint Solver (LINCS)^60^ and particle mesh Ewald^61^ was used to treat long-range electrostatic interactions. The structures obtained after an initial equilibration for 1 ns at constant temperature and pressure and with constraints on the heavy atom positions were used as the starting conformation for the distributed MD simulations. Production MD simulations were carried out at constant temperature and pressure of 298 K and 1 atm respectively, with a time step of 2 fs. The Amber99sb-ildn (ref. 62) force field was used for protein and ions. Distributed MD simulations were performed using GROMACS^63^ on the Folding@home^64^ computing platform. For the first set of simulations, 5,000 simulations were started from the two structures (2,500 each) for a total aggregate simulation time of 200 μs. Adaptive sampling algorithms^65^ based on an MSM built from the initial round of sampling were used to improve the sampling the conformational landscape of apo CaM. Specifically, the initial structures (500 states with lowest population) for adaptive sampling rounds were chosen from the 2,000 state MSM built using RMSD metric and the simulation data from the first round. In all, we performed a total of 12,184 simulations with a total duration of 455 μs. Trajectory snapshots were recorded every 100 ps. The distribution of total number of simulations versus the simulation length for apo CaM is shown in Supplementary Fig. 16a.

### Simulation details for holo-CaM

The starting structure for the holo-CaM was taken from PDB: 1CLL. The force field parameters for the Ca^2+^ ions were taken from the Amber99sb-ildn (ref. 62) force field. The all-atom structures were then solvated in a cubic box with box length of ∼60 Å with TIP3P water molecules, such that water extended at least 10 Å away from the surface of the protein; 12 Na^+^ ions and 4 Cl^−^ ions were added to the system to neutralize the charge. A total of 9,995 distributed MD simulations were performed using GROMACS on the Folding@home platform for a total duration of 256 μs. Simulation protocols employed for holo-CaM were similar to the protocols described above for the apo CaM. The distribution of total number of simulations versus the simulation length for holo-CaM is shown in Supplementary Fig. 16b. Preservation of Ca^2+^-binding site geometry was confirmed by calculating the RMSD of the Ca^2+^-ligating residues between the holo-crystal structure and each conformation weighted by its MSM probability (Supplementary Fig. 17).

### MSM construction

The theoretical framework underlying MSMs has been detailed extensively elsewhere^22^ so will briefly be summarized herein. MSMs are an application of discrete-space master equations and describe the kinetic network that underlies a particular partitioning of phase space. The phase space explored by biomolecular systems can be discretized by dimensionality reduction along specific order parameters. In this study, we discretized phase space using tICA^29^, which is a variant of principal component analysis that defines a kinetically motivated, projection-based distance metric. The tICA method computes the time-lag correlation matrix, whose eigenvectors represent linear combinations of the most slowly decorrelating degrees of freedom in a system. To apply the tICA method to C-CaM, we chose the degrees of freedom to correspond to the minimum distances in the contact map between the heavy atoms of all residue pairs separated by at least three residues. Representing each conformation as a vector of its pairwise residue distances and projecting these vector representations onto tIC components (eigenvectors of the time-lag correlation matrix) mapped the system to a reduced dimensionality space. The distance between conformations |*A*〉 and |*B*〉 in this reduced tIC space was then calculated by


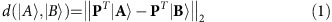


where *P*^*T*^|*X* denotes the projection of the vector representation of conformation *X* onto a *d* × *N* matrix *P*, whose columns are the *N* slowest tIC components and row-length is determined by the size of the contact map, and ||·||_2_ is the *N*-dimensional Euclidean norm. For apo- and holo-C-CaM, the time-lag correlation matrices were calculated with a delta time of 40 ns, and the reduced phase space was computed by projection onto the slowest 20 tICs and clustered into 100 states using the k-centres algorithm.

A kinetic network describing the rates of transitions in this partitioned space was then constructed by computing the transition probability matrix at variable lag-times. The transition probability matrix, *T*(*τ*), contains the probability of transitioning from state *i* to state *j* in the time interval *τ*, obtained by counting the number of transitions, *n*_*ij*_, observed between time *t* and *t*+*τ* and then normalizing to the sum of all transitions from state *i*. Detailed balance is enforced by symmetrizing the transition probability matrix by the maximum likelihood estimate. The first eigenvector of the transition probability matrix corresponds to the equilibrium distribution; subsequent eigenvectors correspond to dynamic processes in the system. Their eigenvalues, *μ*, are related to the implied timescales, 1/*k*, of these dynamic processes:


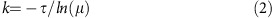


Examining the slowest eigenvalues as a function of lag-time for convergence behaviour identifies a Markovian (memoryless) lag-time. MSMs were constructed at these lag-times (20 and 15 ns for apo- and holo-C-CaM, respectively). Transition Path Theory, which has been outlined elsewhere^66^, was used to determine the pathways and their associated fluxes between specific states. Conformational landscapes were generated by summing over the microstates of each model using the following equation:


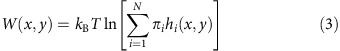


where *N* is the number of microstates, *π*_*i*_ is the equilibrium probability of state *i* and *h*_*i*_(*x*,*y*) is the normalized histogram of the order parameters *x* and *y* restricted to microstate *i*. MSMs were built using the MSMbuilder^67^ software.

### Generation and analysis of MSM trajectories

Representative trajectories that recapitulate the MSM can be generated by applying a kinetic Monte Carlo sampling scheme to the transition probability matrix^19^. Given an MSM trajectory in state *i* at time *t*, the subsequent state visited at *t*+*τ* (a period of one lag-time) was determined by sampling from the multinomial distribution given by *T*_*i*_. For each frame of the resulting trajectory, a random conformation belonging to the relevant state was selected and from this a time-series of the observables was calculated. For both apo- and holo-C-CaM, the trajectories were started from the most populated state at equilibrium.

Comparison with the following structures from the Protein Data Bank was made in Figs 3,6: 1CKK, 1CM1, 1G4Y, 1IQ5, 1IWQ, 1K90, 1NIW, 1NID, 1XA5, 1YRT, 2BBM, 2F3Y, 2HQW, 2KDU, 2M55, 2MG5, 2MGU, 2W73, 2YGG, 3BXL, 3G43, 3SJQ, 4BYF and 4EHQ. In each case, only residues E82-A147 from a single chain of CaM were analysed, and missing C-terminal residues were modelled in using the Crystallographic Object-Oriented Toolkit (Coot, v 0.7.1) (ref. 68) or Modeller, v 9.14 (ref. 69) where necessary.

Macrostate models of the most populated binding interfaces were built by using hierarchical clustering^70^ to partition each MSM trajectory into a five state model based on RMSD of the following residues that comprise the hydrophobic binding interface: Phe89, Phe92, Ile100, Leu105, Val108, Met109, Leu112, Leu116, Met124, Ala128, Val136, Phe141, Met144 and Met145.

### Data availability

Simulation data for apo and holo C-CaM are available at the Stanford Data Repository: https://purl.stanford.edu/zw177zm0384.

## Additional information

**How to cite this article**: Shukla, D. *et al*. Conformational heterogeneity of the calmodulin binding interface. *Nat. Commun.* 7:10910 doi: 10.1038/ncomms10910 (2016).

## Supplementary Material

Supplementary Information Supplementary Figures 1-17, Supplementary Tables 1-3, Supplementary Methods and Supplementary References

## References

[b1] Weinstein H. & Mehler E. L. Ca^2+^-binding and structural dynamics in the functions of calmodulin. Annu. Rev. Physiol. 56, 213–236 (1994). 8010740 10.1146/annurev.ph.56.030194.001241

[b2] Clapham D. E. Calcium signaling. Cell 131, 1047–1058 (2007). 18083096 10.1016/j.cell.2007.11.028

[b3] Berridge M. J., Lipp P. & Bootman M. D. The versatility and universality of calcium signalling. Nat. Rev. Mol. Cell Biol. 1, 11–21 (2000). 11413485 10.1038/35036035

[b4] Chin D. & Means A. R. Calmodulin: a prototypical calcium sensor. Trends Cell Biol. 10, 322–328 (2000). 10884684 10.1016/s0962-8924(00)01800-6

[b5] Zhang M. . Structural basis for calmodulin as a dynamic calcium sensor. Structure 20, 911–923 (2012). 22579256 10.1016/j.str.2012.03.019 PMC3372094

[b6] Babu Y. S. . Three-dimensional structure of calmodulin. Nature 315, 37–40 (1984). 3990807 10.1038/315037a0

[b7] Linse S., Helmersson A. & Forsén S. Calcium binding to calmodulin and its globular domains. J. Biol. Chem. 266, 8050–8054 (1991). 1902469

[b8] Heidorn D. B. & Trewhella J. Comparison of the crystal and solution structures of calmodulin and troponin C. Biochemistry 27, 909–915 (1988). 3365370 10.1021/bi00403a011

[b9] Tjandra N., Kuboniwa H., Ren H. & Bax A. Rotational dynamics of calcium-free calmodulin studied by 15N-NMR relaxation measurements. Eur. J. Biochem. 230, 1014–1024 (1995). 7601131 10.1111/j.1432-1033.1995.tb20650.x

[b10] Finn B. E. . Calcium-induced structural changes and domain autonomy in calmodulin. Nat. Struct. Mol. Biol. 2, 777–783 (1995). 10.1038/nsb0995-777 7552749

[b11] Hoeflich K. P. & Ikura M. Calmodulin in action: diversity in target recognition and activation mechanisms. Cell 108, 739–742 (2002). 11955428 10.1016/s0092-8674(02)00682-7

[b12] Piazza M., Taiakina V., Guillemette S. R., Guillemette J. G. & Dieckmann T. Solution structure of calmodulin bound to the target peptide of endothelial nitric oxide synthase phosphorylated at Thr495. Biochemistry 53, 1241–1249 (2014). 24495081 10.1021/bi401466s

[b13] Yamauchi E., Nakatsu T., Matsubara M., Kato H. & Taniguchi H. Crystal structure of a MARCKS peptide containing the calmodulin-binding domain in complex with Ca^2+^-calmodulin. Nat. Struct. Mol. Biol. 10, 226–231 (2003). 10.1038/nsb900 12577052

[b14] Chen Y.-G. & Hummer G. Slow conformational dynamics and unfolding of the calmodulin C-terminal domain. J. Am. Chem. Soc. 129, 2414–2415 (2007). 17290995 10.1021/ja067791a

[b15] Tripathi S. & Portman J. J. Inherent flexibility determines the transition mechanisms of the EF-hands of calmodulin. Proc. Natl. Acad. Sci. USA 106, 2104–2109 (2009). 19190183 10.1073/pnas.0806872106 PMC2650115

[b16] Stigler J. & Rief M. Calcium-dependent folding of single calmodulin molecules. Proc. Natl. Acad. Sci. USA 109, 17814–17819 (2012). 22753517 10.1073/pnas.1201801109 PMC3497792

[b17] Rabl C.-R., Martin S. R., Neumann E. & Bayley P. M. Temperature jump kinetic study of the stability of apo-calmodulin. Biophys. Chem. 101, 553–564 (2002). 12488026 10.1016/s0301-4622(02)00150-3

[b18] Kohlhoff K. . Cloud-based simulations on Google Exacycle reveal ligand modulation of GPCR activation pathways. Nat. Chem. 6, 15–21 (2014). 24345941 10.1038/nchem.1821 PMC3923464

[b19] Shukla D., Meng Y., Roux B. & Pande V. S. Activation pathway of Src kinase reveals intermediate states as targets for drug design. Nat. Commun. 5, 3397 (2014). 24584478 10.1038/ncomms4397 PMC4465921

[b20] Lawrenz M., Shukla D. & Pande V. S. Cloud computing approaches for prediction of ligand binding poses and pathways. Sci. Rep. 5, 7918 (2015). 25608737 10.1038/srep07918 PMC4302315

[b21] Lane T. J., Shukla D., Beauchamp K. A. & Pande V. S. To milliseconds and beyond: challenges in the simulation of protein folding. Curr. Opin. Struct. Biol. 23, 58–65 (2013). 23237705 10.1016/j.sbi.2012.11.002 PMC3673555

[b22] Shukla D., Hernndez C. X., Weber J. K. & Pande V. S. Markov state models provide insights into dynamic modulation of protein function. Acc. Chem. Res. 48, 414–422 (2015). 25625937 10.1021/ar5002999 PMC4333613

[b23] Komeiji Y., Ueno Y. & Uebayasi M. Molecular dynamics simulations revealed Ca^2+^-dependent conformational change of calmodulin. FEBS Lett. 521, 133–139 (2002). 12067741 10.1016/s0014-5793(02)02853-3

[b24] Shepherd C. M. & Vogel H. J. A molecular dynamics study of Ca^2+^-calmodulin: Evidence of interdomain coupling and structural collapse on the nanosecond timescale. Biophys. J. 87, 780–791 (2004). 15298887 10.1529/biophysj.103.033266 PMC1304488

[b25] Hait W. N. & Lazo J. Calmodulin: a potential target for cancer chemotherapeutic agents. J. Clin. Oncol. 4, 994–1012 (1986). 2423656 10.1200/JCO.1986.4.6.994

[b26] O'Day D. H. & Myre M. A. Calmodulin-binding domains in Alzheimer's disease proteins: extending the calcium hypothesis. Biochem. Biophys. Res. Commun. 320, 1051–1054 (2004). 15249195 10.1016/j.bbrc.2004.06.070

[b27] Kuboniwa H. . Solution structure of calcium-free calmodulin. Nat. Struct. Biol 2, 768–776 (1995). 7552748 10.1038/nsb0995-768

[b28] Chattopadhyaya R., Meador W. E., Means A. R. & Quiocho F. A. Calmodulin structure refined at 1.7 Å resolution. J. Mol. Biol. 228, 1177–1192 (1992). 1474585 10.1016/0022-2836(92)90324-d

[b29] Schwantes C. R. & Pande V. S. Improvements in Markov state model construction reveal many non-native interactions in the folding of NTL9. J. Chem. Theory Comput. 9, 2000–2009 (2013). 23750122 10.1021/ct300878a PMC3673732

[b30] Malmendal A., Evenäs J., Forsén S. & Akke M. Structural dynamics in the C-terminal domain of calmodulin at low calcium levels. J. Mol. Biol. 293, 883–899 (1999). 10543974 10.1006/jmbi.1999.3188

[b31] Marlow M. S., Dogan J., Frederick K. K., Valentine K. G. & Wand A. J. The role of conformational entropy in molecular recognition by calmodulin. Nat. Chem. Biol. 6, 352–358 (2010). 20383153 10.1038/nchembio.347 PMC3050676

[b32] Bowman G. R. & Geissler P. L. Extensive conformational heterogeneity within protein cores. J. Phys. Chem. B 118, 6417–6423 (2014). 24564338 10.1021/jp4105823 PMC4065209

[b33] Evenäs J., Forsn S., Malmendal A. & Akke M. Backbone dynamics and energetics of a calmodulin domain mutant exchanging between closed and open conformations. J. Mol. Biol. 289, 603–617 (1999). 10356332 10.1006/jmbi.1999.2770

[b34] Evenäs J., Malmendal A. & Akke M. Dynamics of the transition between open and closed conformations in a calmodulin C-terminal domain mutant. Structure. 9, 185–195 (2001). 11286885 10.1016/s0969-2126(01)00575-5

[b35] Weininger U. . Protein conformational exchange measured by 1H R1pÂrelaxation dispersion of methyl groups. J. Biomol. NMR 57, 47–55 (2013). 23904100 10.1007/s10858-013-9764-4

[b36] Lundström P. & Akke M. Quantitative analysis of conformational exchange contributions to 1Hâ̂'15N multiple-quantum relaxation using field-dependent measurements. Time scale and structural characterization of exchange in a calmodulin C-terminal domain mutant. J. Am. Chem. Soc. 126, 928–935 (2004). 14733570 10.1021/ja037529r

[b37] Lundström P. & Akke M. Microsecond protein dynamics measured by 13C rotating-frame spin relaxation. Chembiochem. 6, 1685–1692 (2005). 16028301 10.1002/cbic.200500086

[b38] Cook W. J., Walter L. J. & Walter M. R. Drug binding by calmodulin: crystal structure of a calmodulin-trifluoperazine complex. Biochemistry 33, 15259–15265 (1994). 7803388 10.1021/bi00255a006

[b39] Fallon J. L. . Crystal structure of dimeric cardiac L-type calcium channel regulatory domains bridged by Ca^2+^ calmodulins. Proc. Natl. Acad. Sci. USA 106, 5135–5140 (2009). 19279214 10.1073/pnas.0807487106 PMC2654391

[b40] Masino L., Martin S. R. & Bayley P. M. Ligand binding and thermodynamic stability of a multidomain protein, calmodulin. Protein Sci. 9, 1519–1529 (2000). 10975573 10.1110/ps.9.8.1519 PMC2144730

[b41] Lindorff-Larsen K. . Systematic validation of protein force fields against experimental data. PLoS ONE 7, e32131 (2012). 22384157 10.1371/journal.pone.0032131 PMC3285199

[b42] Münnich S., Taft M. H. & Manstein D. J. Crystal structure of human myosin 1c –- the motor in GLUT4 exocytosis: Implications for Ca^2+^ regulation and 14-3-3 binding. J. Mol. Biol. 426, 2070–2081 (2014). 24636949 10.1016/j.jmb.2014.03.004

[b43] Schumacher M. A., Rivard A. F., Bächinger H. P. & Adelman J. P. Structure of the gating domain of a Ca^2+^-activated K^+^ channel complexed with Ca^2+^/calmodulin. Nature 410, 1120–1124 (2001). 11323678 10.1038/35074145

[b44] McClendon C., Friedland G., Mobley D., Amirkhani H. & Jacobson M. Quantifying correlations between allosteric sites in thermodynamic ensembles. J. Chem. Theory Comput. 5, 2486–2502 (2009). 20161451 10.1021/ct9001812 PMC2790287

[b45] Horváth I. . The structure of the complex of calmodulin with KAR-2: a novel mode of binding explains the unique pharmacology of the drug. J. Biol. Chem. 280, 8266–8274 (2005). 15596444 10.1074/jbc.M410353200

[b46] Vanatta D. K., Shukla D., Lawrenz M. & Pande V. S. A network of molecular switches controls the activation of the two-component response regulator ntrc. Nat. Commun. 6, 7283 (2015). 26073186 10.1038/ncomms8283

[b47] Shukla D., Lawrenz M. & Pande V. S. Elucidating ligand-modulated conformational landscape of gpcrs using cloud-computing approaches. Methods Enzymol. 557, 551–572 (2015). 25950981 10.1016/bs.mie.2014.12.007

[b48] Dyson H. J. & Wright P. E. Intrinsically unstructured proteins and their functions. Nat. Rev. Mol. Cell Biol. 6, 197–208 (2005). 15738986 10.1038/nrm1589

[b49] Fiorin G., Pastore A., Carloni P. & Parrinello M. Using metadynamics to understand the mechanism of calmodulin/target recognition at atomic detail. Biophys. J. 91, 2768–2777 (2006). 16877506 10.1529/biophysj.106.086611 PMC1578468

[b50] Hoang J. & Prosser R. S. Conformational selection and functional dynamics of calmodulin: a (19)F nuclear magnetic resonance study. Biochemistry 53, 5727–5736 (2014). 25148136 10.1021/bi500679c

[b51] Peersen O. B., Madsen T. S. & Falke J. J. Intermolecular tuning of calmodulin by target peptides and proteins: differential effects on Ca^2+^ binding and implications for kinase activation. Protein Sci. 6, 794–807 (1997). 9098889 10.1002/pro.5560060406 PMC2144748

[b52] Aoyagi M., Arvai A. S., Tainer J. A. & Getzoff E. D. Structural basis for endothelial nitric oxide synthase binding to calmodulin. EMBO J. 22, 766–775 (2003). 12574113 10.1093/emboj/cdg078 PMC145438

[b53] Liu Y. . Crystal structure of calmodulin binding domain of Orai1 in complex with Ca^2+^-calmodulin displays a unique binding mode. J. Biol. Chem. 287, 43030–43041 (2012). 23109337 10.1074/jbc.M112.380964 PMC3522297

[b54] Best R. B. & Hummer G. Optimized molecular dynamics force fields applied to the helix-coil transition of polypeptides. J. Phys. Chem. B 113, 9004–9015 (2009). 19514729 10.1021/jp901540t PMC3115786

[b55] Best R. B., Zheng W. & Mittal J. Balanced protein-water interactions improve properties of disordered proteins and non-specific protein association. J. Chem. Theory Comput. 10, 5113–5124 (2014). 25400522 10.1021/ct500569b PMC4230380

[b56] Beauchamp K. A., Lin Y. S., Das R. & Pande V. S. Are protein force fields getting better? a systematic benchmark on 524 diverse NMR measurements. J. Chem. Theory Comput. 8, 1409–1414 (2012). 22754404 10.1021/ct2007814 PMC3383641

[b57] DeMaria C. D., Soong T. W., Alseikhan B. A., Alvania R. S. & Yue D. T. Calmodulin bifurcates the local Ca^2+^ signal that modulates P/Q-type Ca^2+^ channels. Nature 411, 484–489 (2001). 11373682 10.1038/35078091

[b58] Rodríguez-Castañeda F. . Modular architecture of Munc13/calmodulin complexes: dual regulation by Ca^2+^ and possible function in short-term synaptic plasticity. EMBO J. 29, 680–691 (2010). 20010694 10.1038/emboj.2009.373 PMC2830703

[b59] Jorgensen W., Chandrasekhar J., Madura J., Impey R. & Klein M. Comparison of simple potential functions for simulating liquid water. J. Chem. Phys. 79, 926 (1983).

[b60] Hess B. . LINCS: a linear constraint solver for molecular simulations. J. Comput. Chem. 18, 1463–1472 (1997). 10.1021/ct700200b 26619985

[b61] Darden T., York D. & Pedersen L. Particle mesh Ewald: An Nlog(N) method for Ewald sums in large systems. J. Chem. Phys. 98, 10089 (1993).

[b62] Lindorff-Larsen K. . Improved side-chain torsion potentials for the Amber ff99SB protein force field. Proteins 78, 1950–1958 (2010). 20408171 10.1002/prot.22711 PMC2970904

[b63] Hess B., Kutzner C., van der Spoel D. & Lindahl E. Gromacs 4: algorithms for highly efficient, load-balanced, and scalable molecular simulation. J. Chem. Theory Comput. 4, 435–447 (2008). 26620784 10.1021/ct700301q

[b64] Shirts M. & Pande V. Screen savers of the world unite!. Science 290, 1903–1904 (2000). 17742054 10.1126/science.290.5498.1903

[b65] Bowman G., Ensign D. & Pande V. Enhanced modeling via network theory: Adaptive sampling of Markov state models. J. Chem. Theory Comput. 6, 787–794 (2010). 23626502 10.1021/ct900620b PMC3637129

[b66] Metzner P., Schütte C. & Vanden-Eijnden E. Transition path theory for markov jump processes. Multiscale Model Simul. 7, 1192–1219 (2009).

[b67] Beauchamp K. A. . MSMBuilder2: modeling conformational dynamics on the picosecond to millisecond scale. J. Chem. Theory Comput. 7, 3412–3419 (2011). 22125474 10.1021/ct200463m PMC3224091

[b68] Emsley P., Lohkamp B., Scott W. & Cowtan K. Features and development of Coot. Acta Crystallogr. D Biol. Crystallogr. 66, 486–501 (2010). 20383002 10.1107/S0907444910007493 PMC2852313

[b69] Webb B. & Sali A. . Protein Structure Prediction 1–15Springer (2014).

[b70] Yao Y. . Hierarchical Nyström methods for constructing Markov state models for conformational dynamics. J. Chem. Phys. 138, 174106 (2013). 23656113 10.1063/1.4802007

