# Corrigendum: Conformational heterogeneity of the calmodulin binding interface

**DOI:** 10.1038/ncomms12318

**Published:** 2016-08-10

**Authors:** Diwakar Shukla, Ariana Peck, Vijay S Pande

Nature Communications 7: Article number: 1091010.1038/ncomms10910 (2016); Published: 04 04 2016; Updated: 08 10 2016

In the Methods sections ‘Simulation details for apo CaM' and ‘Simulation details for holo CaM,' the aggregate simulation times for each data set were inadvertently swapped. The correct aggregate simulation times for the apo and holo CaM data sets are 256 and 455 μs, respectively.

A similar error occurs in the figure legend for Supplementary Fig. 16. The figure legend should read: ‘Supplementary Fig. 16: Distribution of simulated trajectory lengths. For the (a) apo and (b) holo CaM systems, 12,184 and 9,995 trajectories were simulated for a total time of 256 and 455 μs, respectively. Trajectories shorter than the lag time at which the MSMs were built were eliminated from subsequent analysis.'

