# Supplementary material for: Conformational heterogeneity of the calmodulin binding interface
Source: Nat Commun. 2016 Apr 4;7:10910. doi: 10.1038/ncomms10910 (PMC4822001; doi:10.1038/ncomms10910)
Supplement: Supplementary Information — Supplementary Figures 1-17, Supplementary Tables 1-3, Supplementary Methods and Supplementary References [file ncomms10910-s1.pdf]

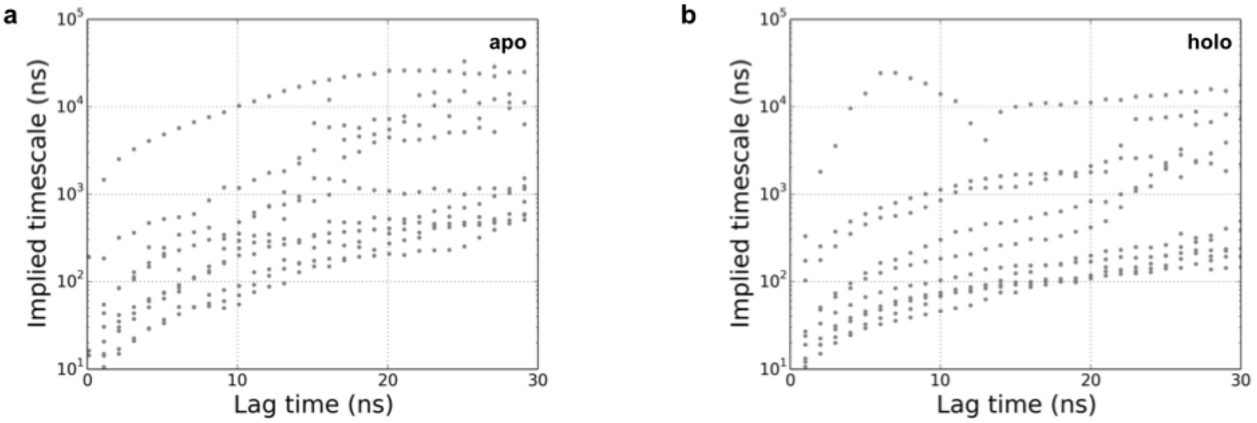

Supplementary Figure 1: Implied timescales of the transition probability matrix. Eigenvalues of the transition probability matrix correspond to the dominant rates of transition in the 100-state model. Shown are the top 10 eigenvalues for the a) apo and b) holo MSMs, which converged at lag times of 20 and 15 ns, respectively.

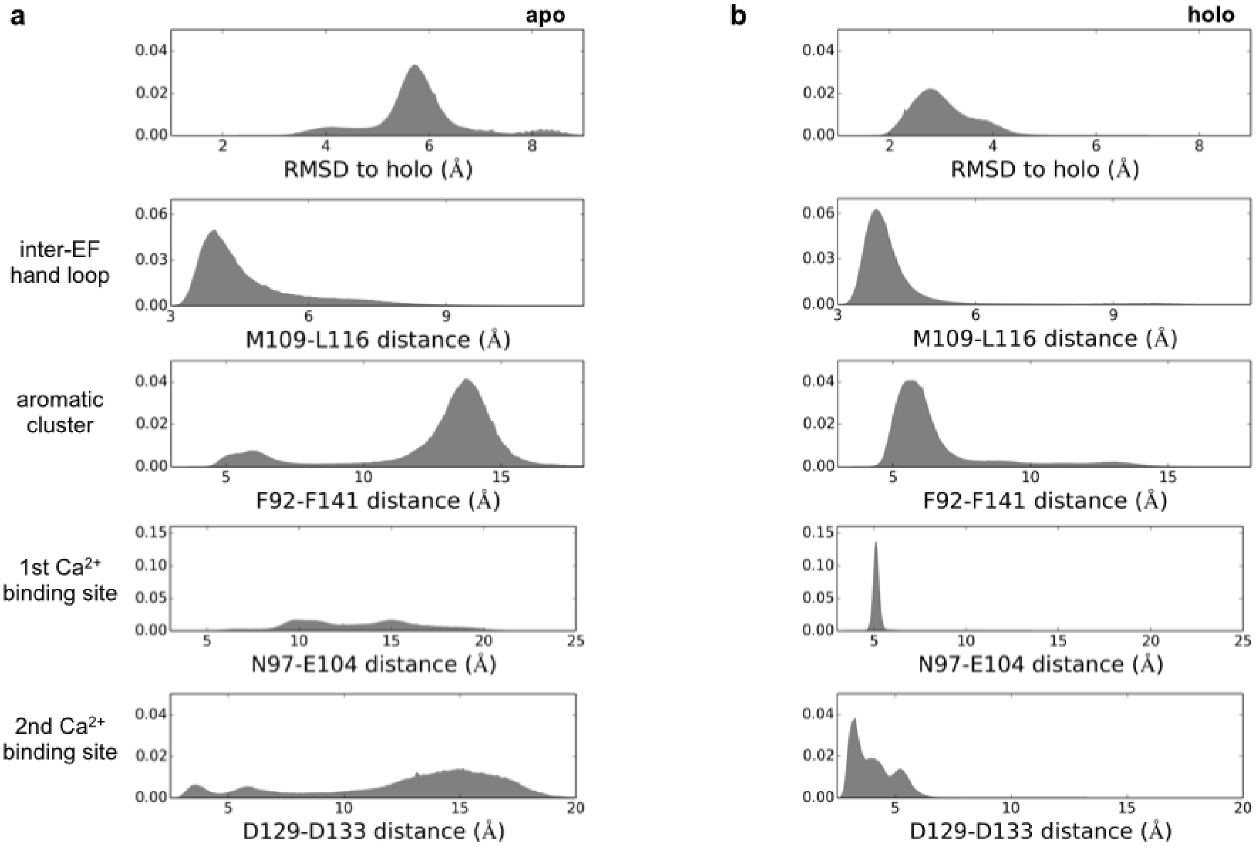

Supplementary Figure 2: Equilibrium populations of apo and holo C-CaM. The first eigenvector of the MSM transition probability matrix describes the equilibrium populations. Each conformation in the (a) apo and (b) holo MSM was weighted by its component of the first eigenvector and projected onto a distance metric.

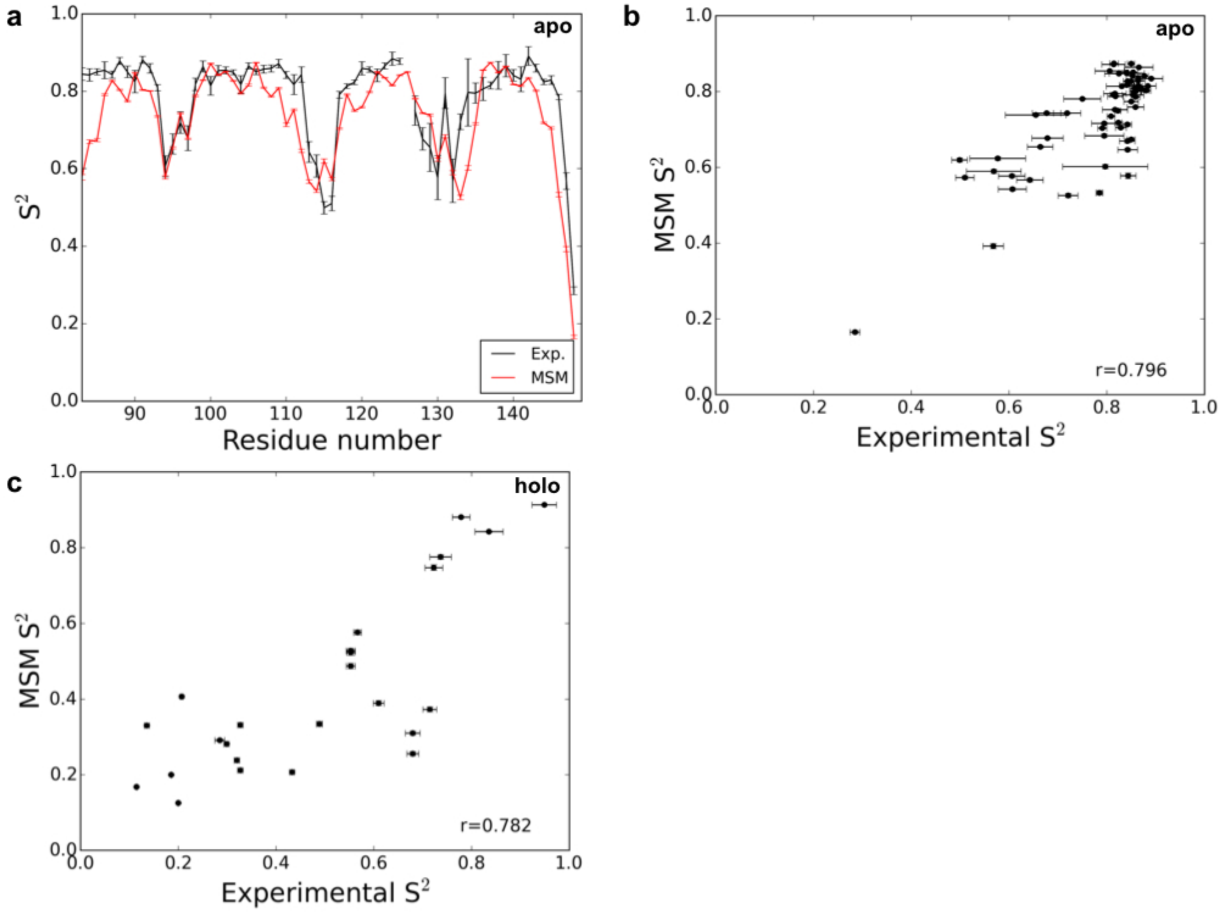

Supplementary Figure 3: Comparison of MSM ensembles to experimental NMR order parameters. NMR S^2 order parameters were estimated from the apo and holo MSM ensembles and compared to experimental values[5,6] for (a, b) backbone amide or (c) side chain methyl groups, respectively. The Pearson correlation coefficient is also noted. Error bars correspond to 95% confidence intervals.

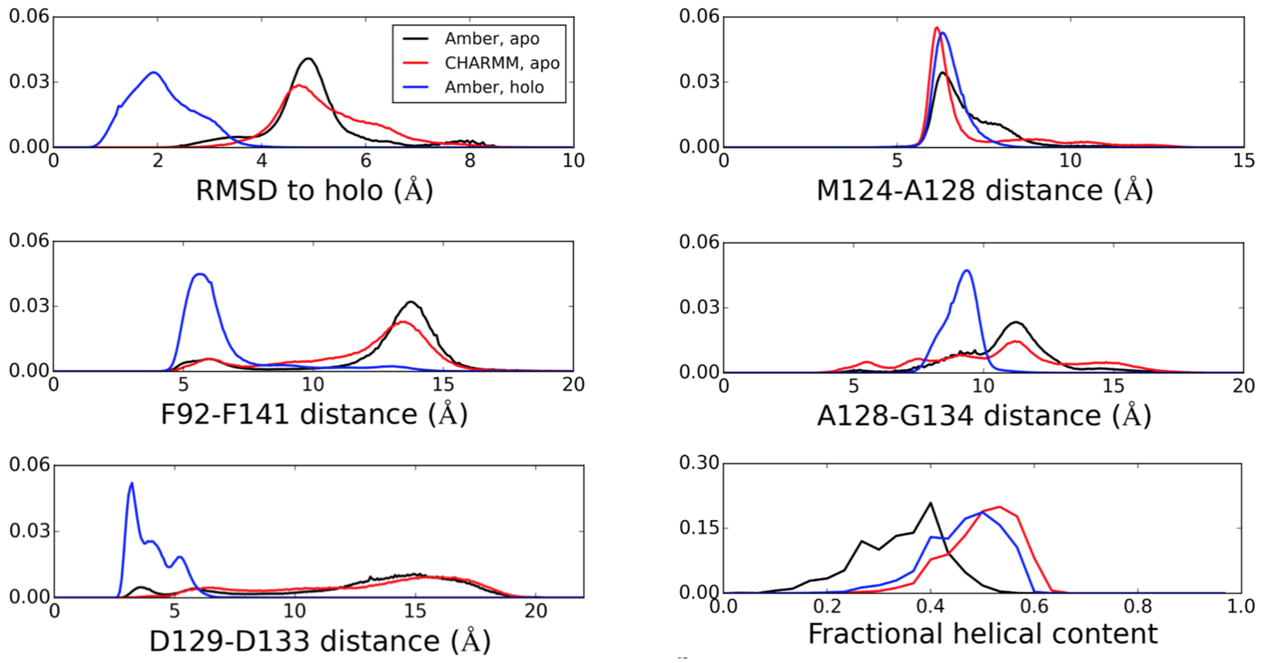

Supplementary Figure 4: Conformational landscape of C-CaM in different force fields. The distributions of conformations explored by apo C-CaM in simulations run in the Amber-99sb-ildn (black) and CHARMM36 (red) force fields are shown; for comparison, distributions for holo C-CaM run in the Amber-99sb-ildn force field (blue) are also shown. Total variation distances between the distributions are listed in Supplementary Tables 2 and 3.

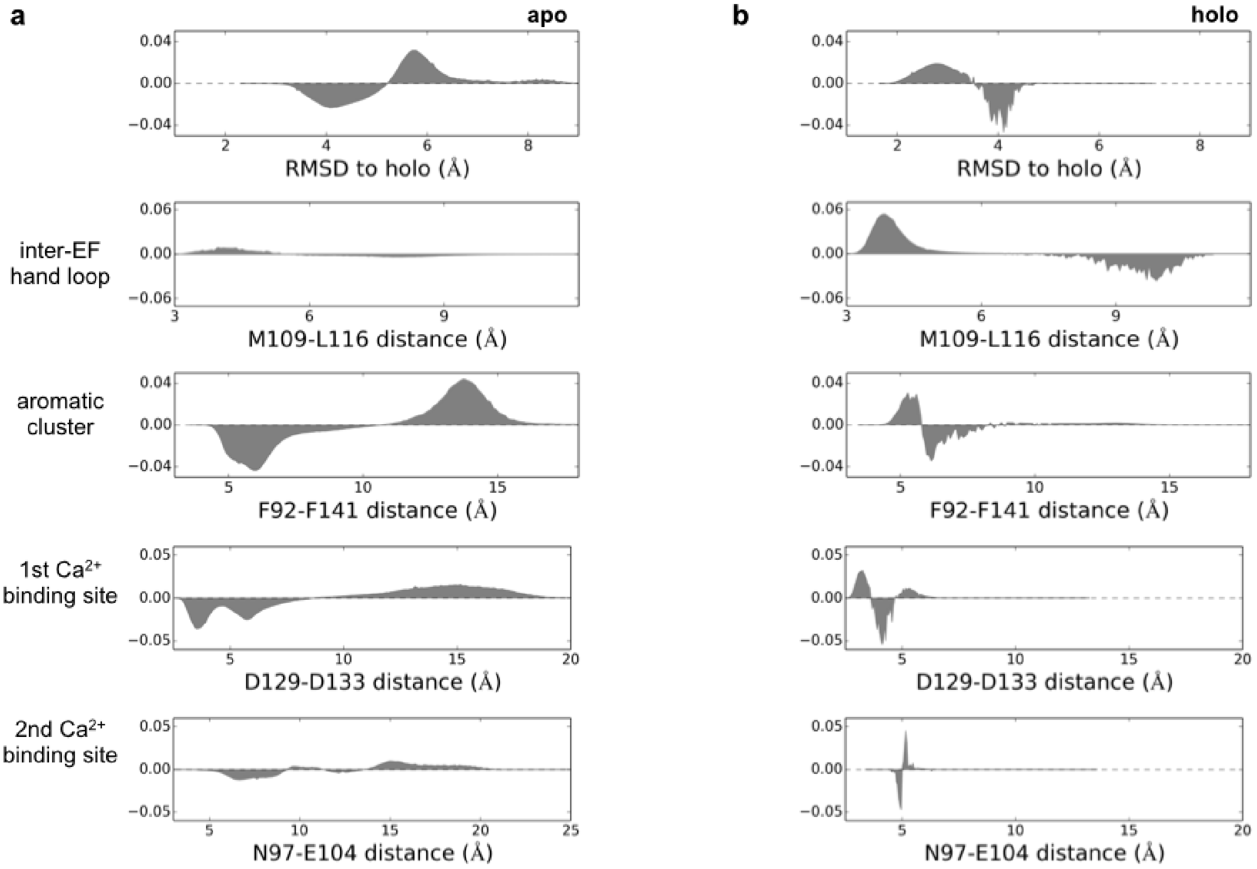

Supplementary Figure 5: Slowest dynamic process of apo and holo C-CaM. The second eigenvector of the MSM transition probability matrix describes the slowest dynamic process. Each conformation in the (a) apo and (b) holo MSM was weighted by its component of the second eigenvector and projected onto a distance metric. In both cases, two-state behavior is evident along different order parameters.

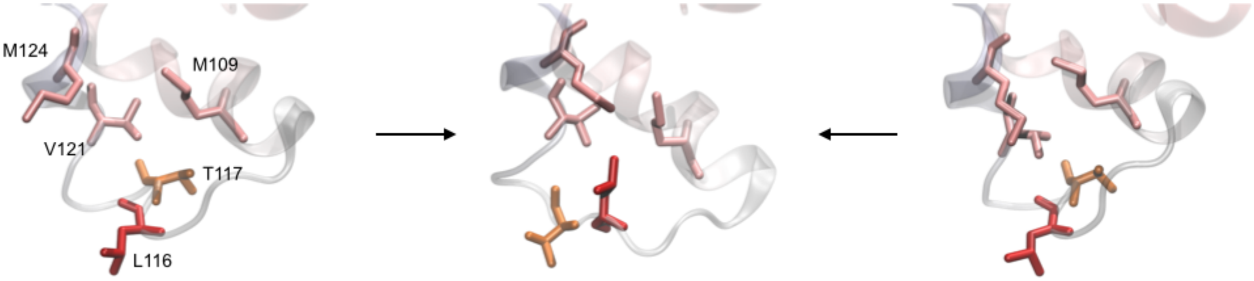

Supplementary Figure 6: Motions in the inter-EF hand loop region of holo C-CaM. Relaxation of the backbone dihedrals in the inter-EF hand loop region is characteristic of the slowest conformational exchange process in the holo regime. Specifically, Leu116 flips from being solvent-exposed to replacing Thr117 in packing against the hydrophobic network formed by Met109, Val121, and Met124. Representative structures are shown for the top two paths, which account for 45% (left) and 15% (right) of flux, respectively.

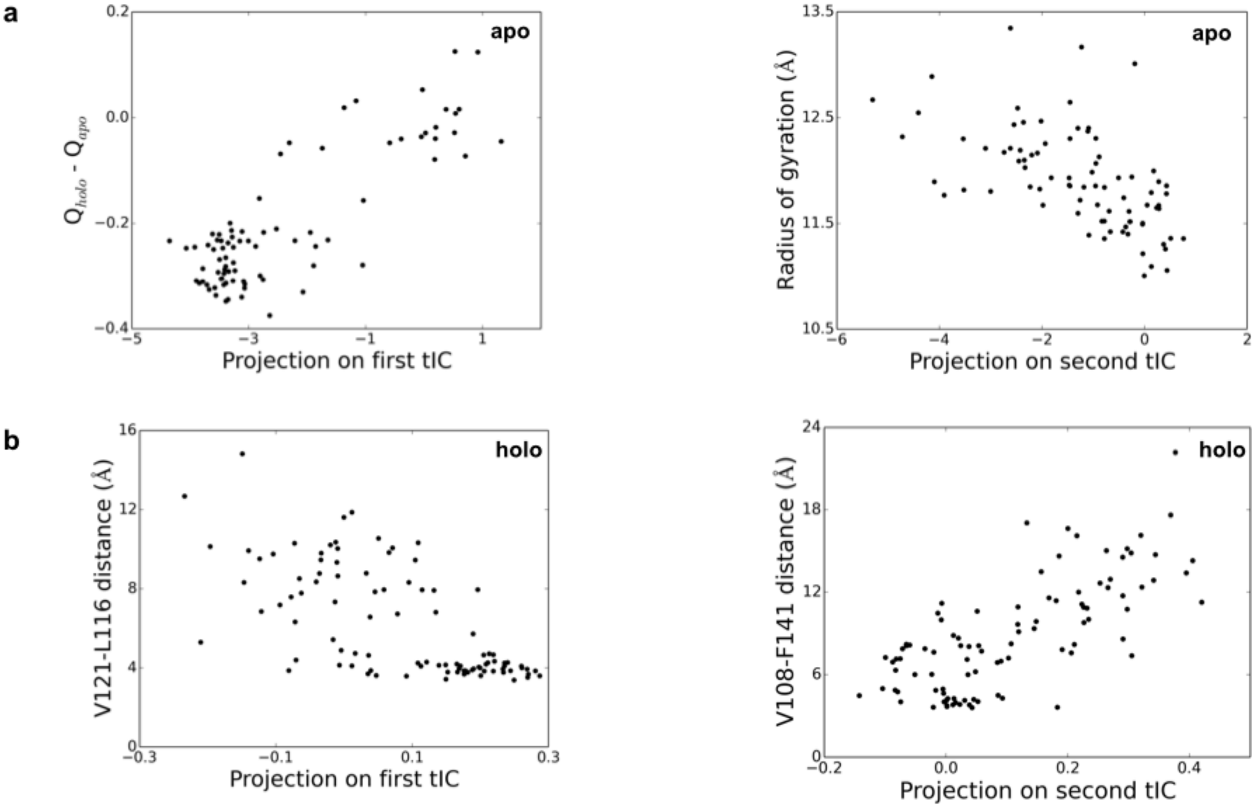

Supplementary Figure 7: Structural rearrangements associated with the dominant tICs. Cluster centers from the (a) apo and (b) holo MSMs were projected onto the first (left) and second (right) tICs, and these values were plotted against various structural metrics. Q is the fraction of native contacts, with 1CFD and 1CLL as the reference apo and holo structures, respectively.

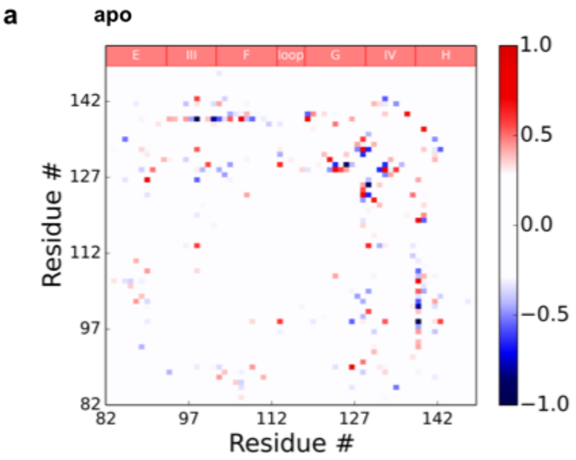

Supplementary Figure 8: Distinct contact maps characterize the slowest decorrelating degrees of freedom in apo and holo C-CaM. The contact maps of the first tIC show that different regions of C-CaM contribute most to the top tIC of (a) apo and (b) holo systems. The components of each tIC were normalized such that the maximum component had a value of 1. Red and blue denote inter-residue distances with positive and negative tIC values, respectively.

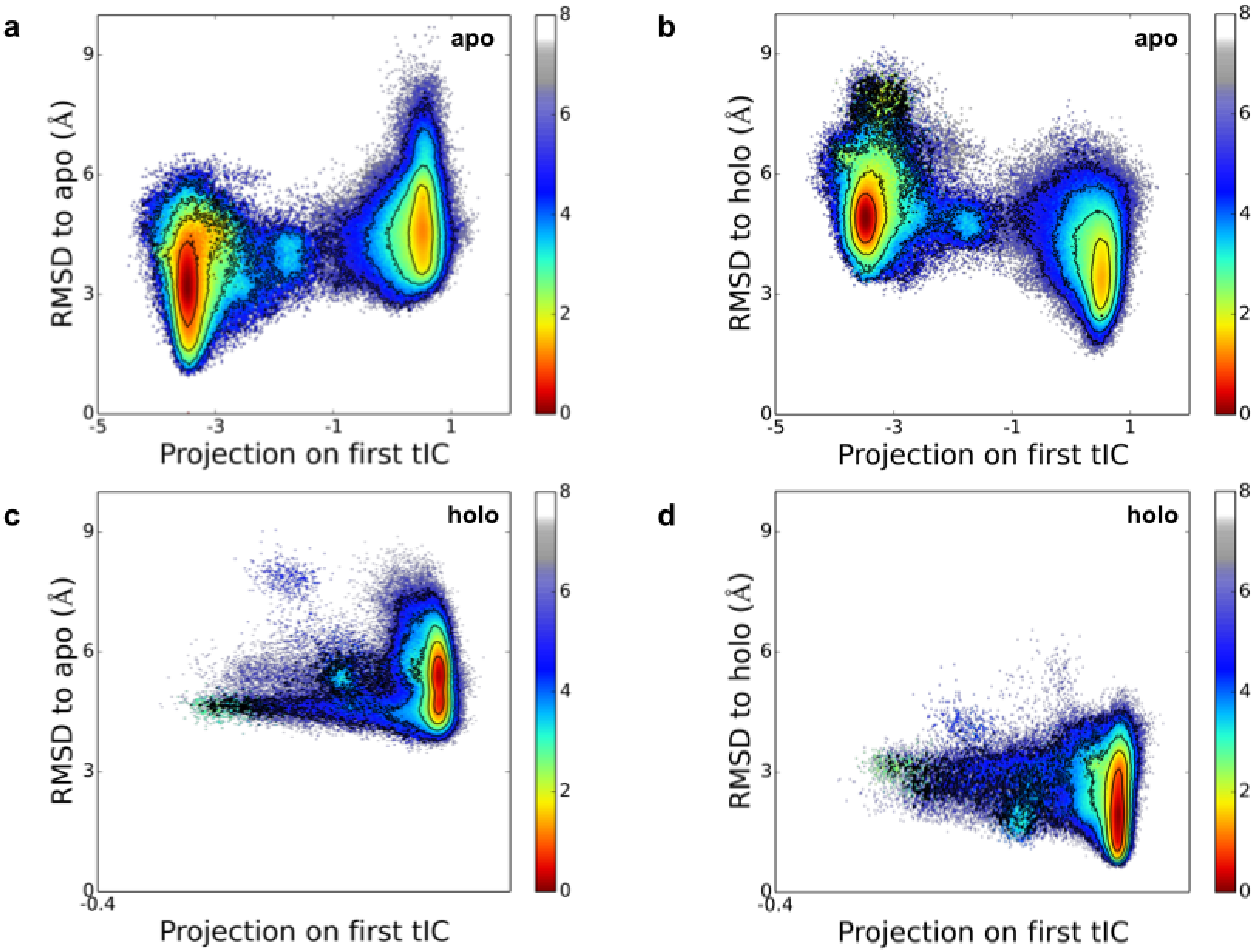

Supplementary Figure 9: The tICs provide a superior separation of the dynamic landscape of C-CaM compared to RMSD. Energy landscapes of apo C-CaM as a function of projection onto the first tIC and RMSD to the (a) apo and (b) holo structures show superior separation along the tIC order parameter as compared to RMSD. These same energy landscapes for holo C-CaM show that RMSD to the (c) apo and (d) holo structures provides no separation for the dynamic processes in holo C-CaM. Conformations were weighted by their MSM probabilities, and free energy values are reported in kcal mol^{-1}. Apo and holo reference structures are 1CFD and 1CLL, respectively.

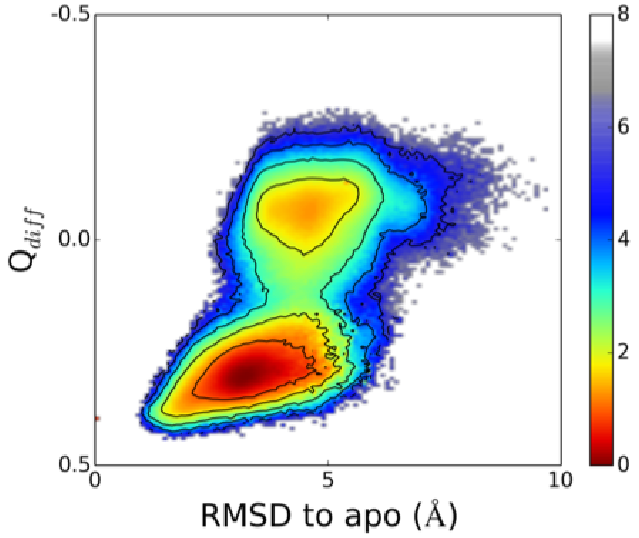

Supplementary Figure 10: Energy landscape of C-CaM according to RMSD and fraction of native contacts. The energy landscape of apo C-CaM was generated by weighting each conformation by its MSM probability and binning by RMSD to the apo structure and Q_{diff}, which denotes the difference between the fractions of native contacts in the apo and holo states. Reference structures for the apo and holo structures were 1CFD and 1CLL, respectively, and free energy values are reported in kcal mol^{-1}.

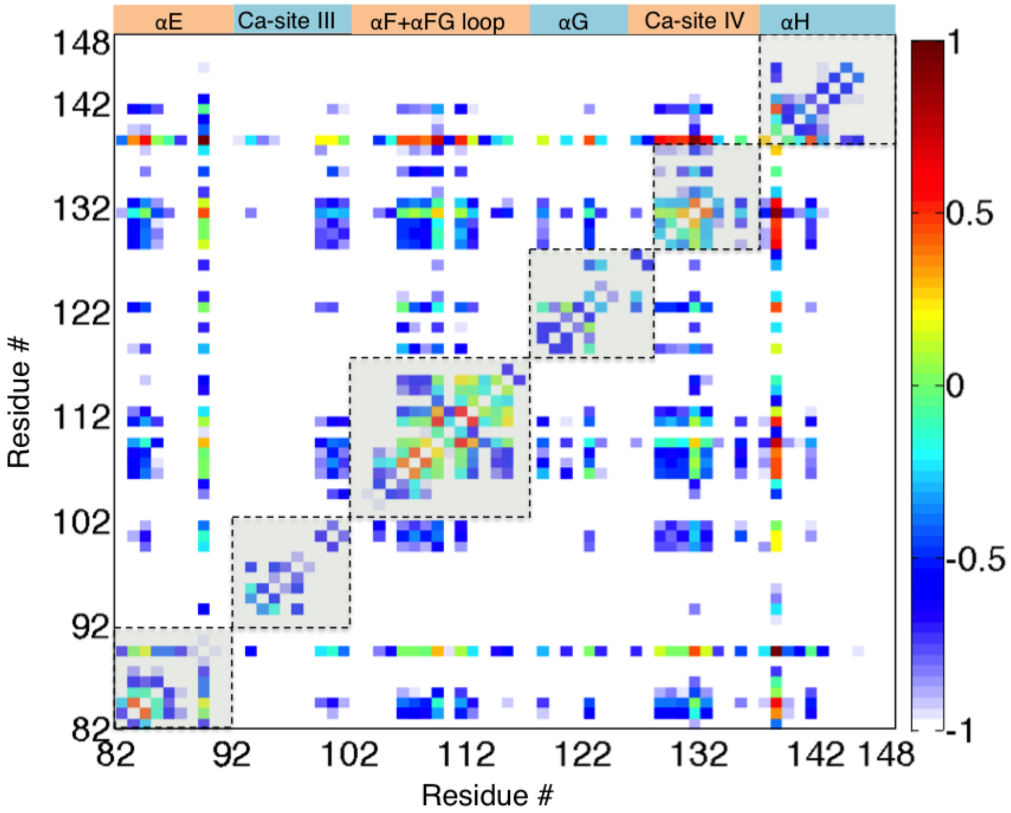

Supplementary Figure 11: Mutual information between residues in apo C-CaM. The most dynamically coupled regions of the protein involve the residues in the αF and the second Ca^{2+}-binding site. The key hydrophobic residues involving in binding of substrate are present on αF. Interestingly, the first Ca^{2+}-binding site is not strongly coupled with any other region of the protein. Colors indicate the log of the mutual information value.

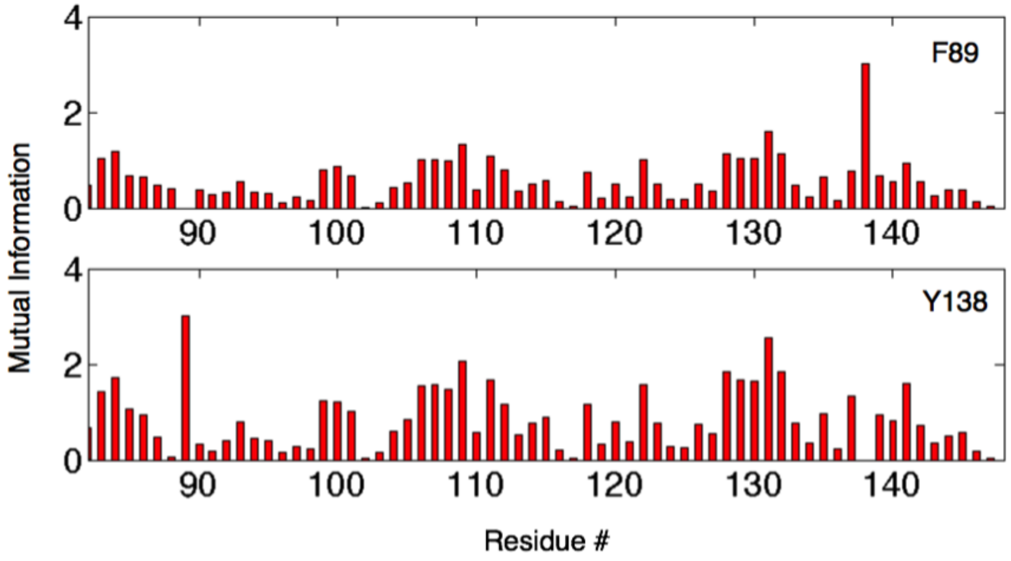

Supplementary Figure 12: Mutual information for key residues in apo C-CaM. Mutual Information values between all the residues and residues (A) Phe89 and (B) Tyr138. These hydrophobic residues are not only dynamically coupled with other residues comprising the hydrophobic binding interface but also to the second Ca^{2+}-binding site.

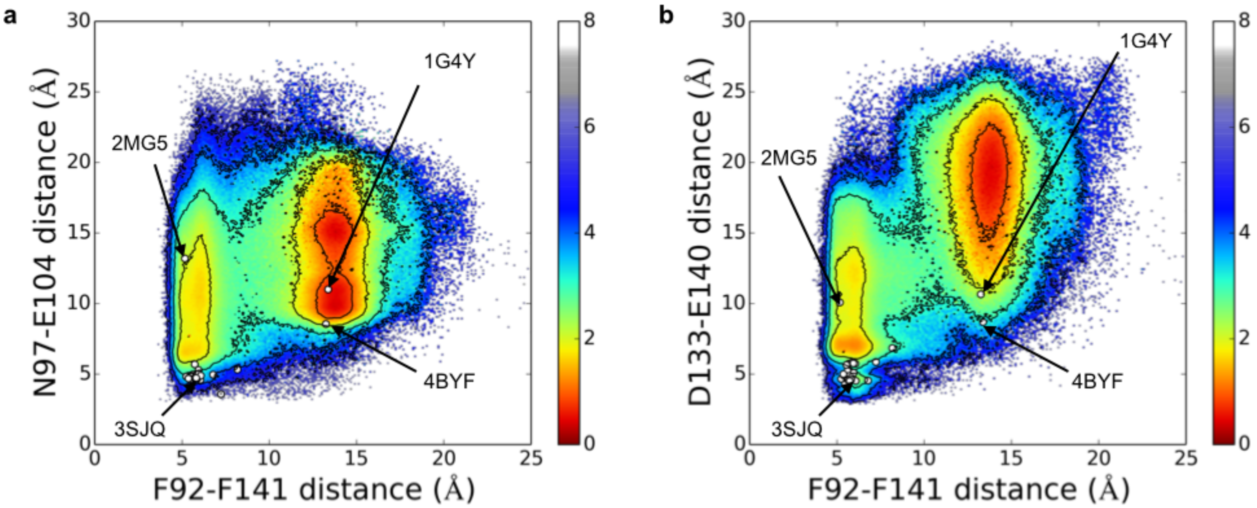

Supplementary Figure 13: The hydrophobic surface and Ca^{2+}-binding sites undergo conformational change in a concerted fashion. The energy landscapes of apo C-CaM as a function of the Phe92-Phe141 distance and the (a) Asn97-Glu104 or (b) Asp133-Glu140 side chain carbonyl distances, which ligate Ca^{2+} in the first and second binding sites, respectively. Conformations were weighted by their MSM probabilities, and free energy values are reported in kcal mol^{-1}. Shown in white are these distances for published structures from the PDB.

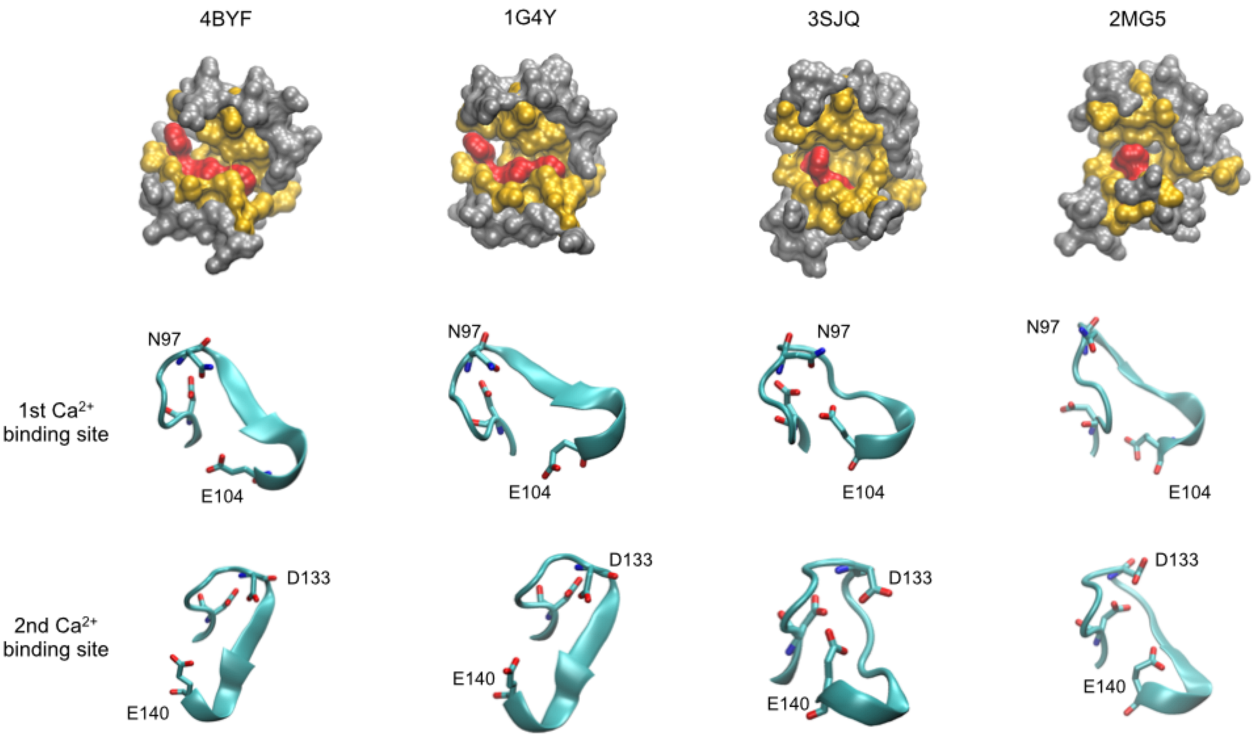

Supplementary Figure 14: Allostery between the hydrophobic surface and Ca^{2+}-binding sites. (*top*) Surface topology of the C-terminal domain from published structures 4BYF, 1G4Y, 3SJQ, and 2MG5 (*left to right*). These correspond to high-resolution structures of full-length CaM in complex with myosin, SK2-a, SK2-b, and phosphorylated nitrogen oxygen synthase, respectively. Phenylalanine, all other hydrophobic, and polar residues are colored red, yellow, and grey, respectively. (*bottom*) Cartoon representation of the first and second Ca^{2+} binding sites. Of these structures only 3SJQ is Ca^{2+}-bound.

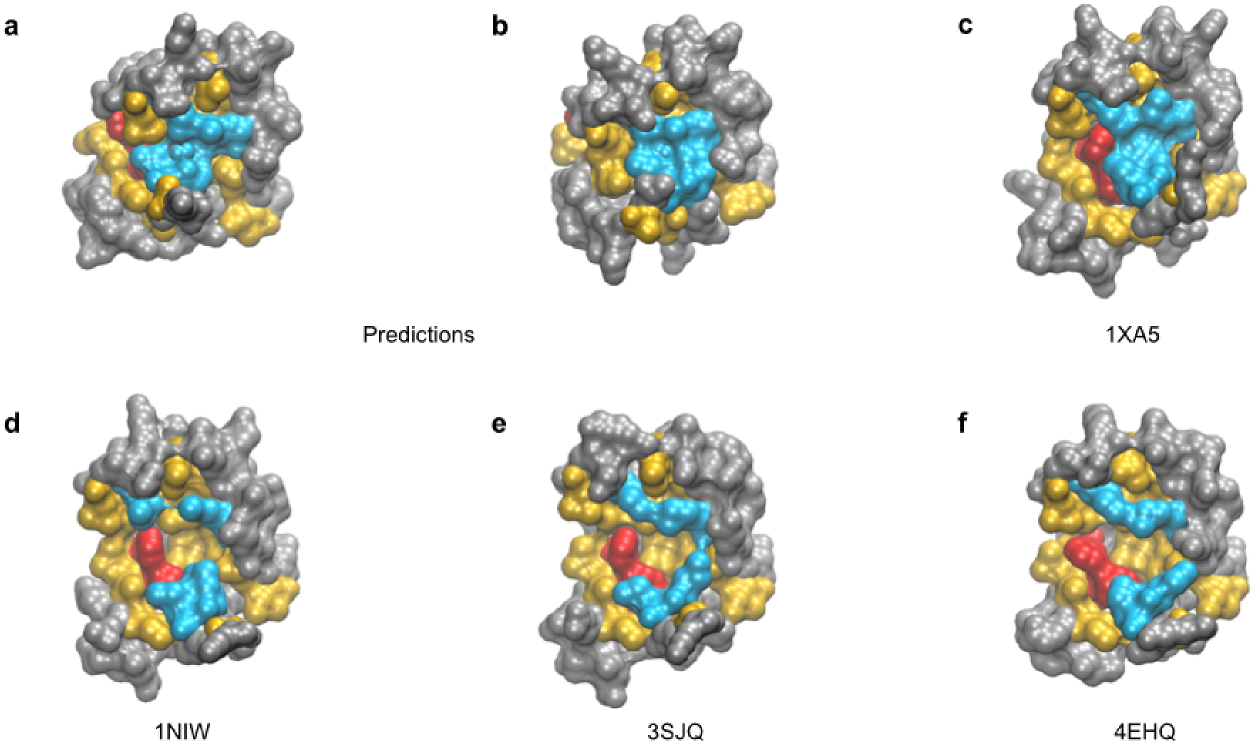

Supplementary Figure 15: Holo C-CaM populates distinct surface topologies. (a) (a, b) Surface representation of the predicted structures with a shallow Met-lined cavity. In terms of the relative positions of the Met and Phe clusters, the exposed binding interface of the C-terminal domain of CaM in complex with the anti-microtublar agent KAR-2 (c) is intermediary between our predicted topologies shown in (a,b) and the canonical Phe-lined pocket topology shown in (d-f), which are derived from high resolution structures of CaM in complex with unphosphorylated nitric oxide synthase, SK2-b, and a Ca^{2+} channel fragment. Phe, Met, all other hydrophobic, and polar residues colored red, cyan, yellow, and grey, respectively. PDB codes for (c-f) are 1XA5, 1NIW, 3SJQ, and 4EHQ, respectively.

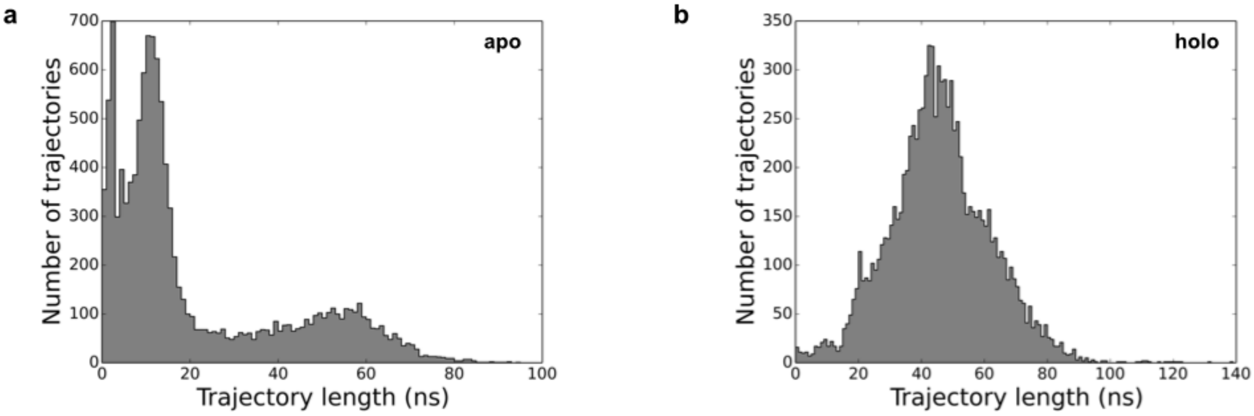

Supplementary Figure 16: Distribution of simulated trajectory lengths. For the (a) apo and (b) holo CaM systems, 9995 and 12,184 trajectories were simulated for a total time of 455 and 256 μs, respectively. Trajectories shorter than the lag time at which the MSMs were built were eliminated from subsequent analysis.

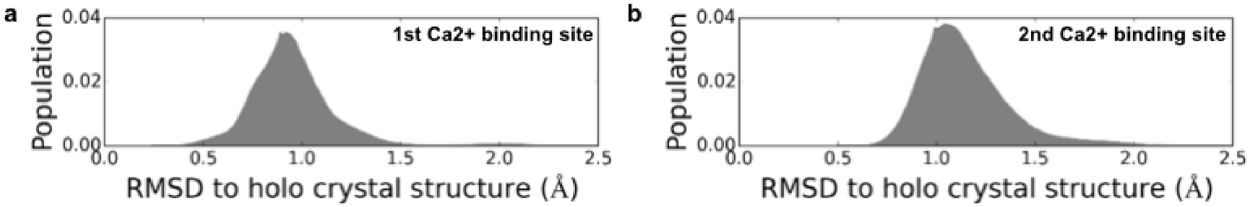

Supplementary Figure 17: Geometry of the Ca^{2+}-binding sites. RMSD of the Ca^{2+}-ligating residues of each conformation in the holo CaM system, weighted by its MSM probability, to the crystal structure (1CLL) for the (a) first and (b) second Ca^{2+}-binding sites.

Residue	MSM	Experimental	Residue	MSM	Experimental
Glu83	0.578 ± 0.007	0.844 ± 0.016	Leu116	0.573 ± 0.004	0.51 ± 0.019
Glu84	0.67 ± 0.005	0.842 ± 0.016	Thr117	0.704 ± 0.002	0.791 ± 0.009
Ile85	0.674 ± 0.004	0.85 ± 0.008	Asp118	0.792 ± 0.002	0.813 ± 0.006
Arg86	0.791 ± 0.003	0.854 ± 0.022	Glu119	0.75 ± 0.002	0.823 ± 0.006
Glu87	0.827 ± 0.002	0.843 ± 0.009	Glu120	0.759 ± 0.002	0.859 ± 0.017
Ala88	0.804 ± 0.002	0.878 ± 0.009	Val121	0.798 ± 0.002	0.856 ± 0.007
Phe89	0.774 ± 0.002	0.85 ± 0.015	Asp122	0.85 ± 0.001	0.841 ± 0.016
Arg90	0.847 ± 0.001	0.825 ± 0.029	Glu123	0.835 ± 0.001	0.865 ± 0.008
Val91	0.803 ± 0.001	0.881 ± 0.009	Met124	0.815 ± 0.001	0.884 ± 0.017
Phe92	0.801 ± 0.001	0.859 ± 0.012	Ile125	0.841 ± 0.001	0.877 ± 0.008
Asp93	0.735 ± 0.002	0.809 ± 0.008	Arg126	0.85 ± 0.002	0.0 ± 0.0
Lys94	0.577 ± 0.004	0.606 ± 0.027	Glu127	0.781 ± 0.002	0.75 ± 0.038
Asp95	0.654 ± 0.003	0.664 ± 0.026	Ala128	0.743 ± 0.002	0.677 ± 0.029
Gly96	0.743 ± 0.002	0.719 ± 0.028	Asp129	0.739 ± 0.003	0.655 ± 0.062
Asn97	0.677 ± 0.002	0.679 ± 0.032	Ile130	0.623 ± 0.004	0.577 ± 0.057
Gly98	0.788 ± 0.002	0.818 ± 0.016	Asp131	0.684 ± 0.004	0.795 ± 0.04
Tyr99	0.829 ± 0.001	0.864 ± 0.014	Gly132	0.59 ± 0.005	0.569 ± 0.056
Ile100	0.871 ± 0.001	0.814 ± 0.024	Asp133	0.526 ± 0.006	0.721 ± 0.02
Ser101	0.842 ± 0.001	0.853 ± 0.013	Gly134	0.602 ± 0.006	0.797 ± 0.087
Ala102	0.851 ± 0.001	0.854 ± 0.009	Gln135	0.716 ± 0.003	0.795 ± 0.026
Ala103	0.827 ± 0.001	0.849 ± 0.006	Val136	0.854 ± 0.001	0.806 ± 0.03
Glu104	0.795 ± 0.002	0.817 ± 0.023	Asn137	0.874 ± 0.001	0.815 ± 0.009
Leu105	0.816 ± 0.003	0.865 ± 0.008	Tyr138	0.848 ± 0.001	0.841 ± 0.036
Arg106	0.874 ± 0.001	0.85 ± 0.007	Glu139	0.864 ± 0.001	0.866 ± 0.029
His107	0.809 ± 0.002	0.857 ± 0.01	Glu140	0.817 ± 0.001	0.841 ± 0.015
Val108	0.786 ± 0.002	0.859 ± 0.009	Phe141	0.814 ± 0.002	0.831 ± 0.032
Met109	0.808 ± 0.002	0.871 ± 0.011	Val142	0.834 ± 0.002	0.891 ± 0.024
Thr110	0.713 ± 0.004	0.842 ± 0.009	Gln143	0.801 ± 0.002	0.86 ± 0.01
Gly111	0.752 ± 0.002	0.817 ± 0.026	Met144	0.718 ± 0.002	0.824 ± 0.007
Leu112	0.646 ± 0.004	0.843 ± 0.02	Met145	0.705 ± 0.003	0.829 ± 0.011
Gly113	0.567 ± 0.005	0.643 ± 0.027	Thr146	0.533 ± 0.006	0.785 ± 0.007
Glu114	0.543 ± 0.004	0.607 ± 0.029	Ala147	0.392 ± 0.006	0.568 ± 0.021
Lys115	0.62 ± 0.005	0.499 ± 0.016	Lys148	0.165 ± 0.004	0.285 ± 0.01

Supplementary Table 1: S^2 order parameters for backbone amides in apo C-CaM. Experimental values are from Malmendal et al[5].

**Residue**	**MSM**	**Experimental**
Ile85δ1	0.238 $\pm$ 0.005	0.32 $\pm$ 0.005
Ile85γ2	0.528 $\pm$ 0.006	0.553 $\pm$ 0.009
Ala88β	0.843 $\pm$ 0.001	0.836 $\pm$ 0.029
Val91γ1	0.310 $\pm$ 0.005	0.68 $\pm$ 0.015
Val91γ2	0.256 $\pm$ 0.005	0.68 $\pm$ 0.012
Ile100γ2	0.776 $\pm$ 0.006	0.737 $\pm$ 0.022
Ala102β	0.914 $\pm$ 0.0	0.949 $\pm$ 0.025
Met109	0.330 $\pm$ 0.002	0.136 $\pm$ 0.005
Leu116δ1	0.291 $\pm$ 0.003	0.285 $\pm$ 0.01
Leu116δ2	0.282 $\pm$ 0.002	0.299 $\pm$ 0.005
Val121γ1	0.524 $\pm$ 0.008	0.553 $\pm$ 0.008
Val121γ2	0.488 $\pm$ 0.007	0.553 $\pm$ 0.009
Met124	0.200 $\pm$ 0.001	0.186 $\pm$ 0.001
Ile125δ1	0.407 $\pm$ 0.004	0.207 $\pm$ 0.003
Ile125γ2	0.577 $\pm$ 0.006	0.567 $\pm$ 0.008
Ala128β	0.881 $\pm$ 0.001	0.779 $\pm$ 0.018
Ile130δ1	0.331 $\pm$ 0.005	0.327 $\pm$ 0.005
Ile130γ2	0.334 $\pm$ 0.006	0.489 $\pm$ 0.006
Val136γ2	0.748 $\pm$ 0.007	0.723 $\pm$ 0.018
Val142γ1	0.373 $\pm$ 0.007	0.715 $\pm$ 0.014
Val142γ1	0.39 $\pm$ 0.006	0.610 $\pm$ 0.011
Met144	0.168 $\pm$ 0.002	0.115 $\pm$ 0.001
Met145	0.126 $\pm$ 0.002	0.200 $\pm$ 0.001
Thr146γ	0.207 $\pm$ 0.004	0.433 $\pm$ 0.005
Ala147β	0.212 $\pm$ 0.004	0.327 $\pm$ 0.005

Supplementary Table 2: S^2 order parameters for side chain methyl groups in holo C-CaM. Experimental values are from Marlow et al[6].

Metric	Amber (apo)-<br>Charmm (apo)	Amber (apo)<br>Amber (holo)	Amber (holo)-<br>Charmm (apo)
RMSD to holo (Å)	0.23	0.92	0.97
F92-F141 (Å)	0.21	0.76	0.72
D129-D131 (Å)	0.16	0.86	0.92
M124-A128 (Å)	0.34	0.32	0.26
A128-G134 (Å)	0.27	0.70	0.71
Helical content	0.72	0.59	0.20

Supplementary Table 3: Total variation distances between the equilibrium distributions of apo or holo C-CaM run in the specified force field.

Supplementary Methods

Simulation details using the CHARMM force field Two conformations from each state of the apo MSM were taken as seeds for additional simulations run using the CHARMM36 force field[1]. Structures were solvated with TIP3P[2] water molecules such that water extended at least 10 Å away from the surface of the protein, and 24 Na^+ ions and 12 Cl^- ions were added to the system to neutralize the charge. Energy minimization for 1000 cycles and heating to 300 K were performed in Amber 14; production MD trajectories were run for a total of 32 μs.

Mutual Information The excess mutual information was computed for all protein torsion angles (backbone dihedrals ϕ, ψ and side chain χ angles (only the first χ angle for proline)) using the entire simulation data for apo C-CaM to capture the correlated motions of residues in an unbiased, statistically robust manner. The following formula was used for the calculation of mutual information between residue pairs[3]:

$$I_{i,j}^{i \neq j} = \sum_{\theta_i} \sum_{\theta_j} \int_0^{2\pi} \int_0^{2\pi} p(\theta_i, \theta_j) \ln \frac{p(\theta_i, \theta_j)}{p(\theta_i) p(\theta_j)} d\theta_i d\theta_j \tag{1}$$

The average of the mutual information computed from 3 iterations of scrambled data was subtracted from the mutual information values computed from the simulation data to filter out correlations that are not statistically significant.

NMR order parameters The NMR order parameter, S^2, describes the bond vector autocorrelation function:

$$S^2 = \lim_{\tau \to \infty} \langle P_2(\vec{\mu}(t_0) \cdot \vec{\mu}(t_0 + \tau)) _{t_0} \rangle \tag{2}$$

where P_2 is the second-order Legendre polynomial, $\vec{\mu}(t_0)$ is the unit vector along a specific bond at time t_0, and $\langle ... \rangle$ indicates the ensemble average[4]. For comparison to relaxation-based order parameters, equation 2 is evaluated at the experimentally-determined molecular tumbling time (5.0 and 8.2 ns for apo and holo CaM, respectively[5,6]). S^2 values were evaluated from all simulation data retained in each MSM; because autocorrelation reduces the effective number of independent samples, the effective number of data points was estimated from:

$$n_{effective} = \frac{n}{\tau_{int}} \tag{3}$$

where n is the number of data points in the autocorrelation calculation and τ_{int} is the estimated integrated autocorrelation time of the time series[7]. Error bars represent the 95% confidence intervals. Order parameters calculated for the apo and holo systems were compared to experimental data for backbone amide collected in the absence of Ca^{2+} and side chain methyl groups[5] collected for Ca^{2+}-saturated CaM[6], respectively.

Native contacts The following expression was used for the calculation of the fraction of native contacts, $Q(X)$, for a conformation X:

$$Q(X) = \frac{1}{\mid S \mid} \sum_{(i,j)\in S} \frac{1}{1 + exp(\beta(r_{ij}(X) - \lambda r_{ij}^{0})} \tag{4}$$

where $r_{ij}(X)$ is the distance between residues i and j in conformation X, r_{ij}^{0} is the distance between residues i and j in the reference conformation (the apo or holo state of C-CaM). The set S represents all pairs of heavy atoms (i, j) belonging to residues θ_i and θ_j such that $\mid \theta_i - \theta_j \mid > 3$ and $r_{ij}^{0} < 4.5$ Å. The values of parameters β and λ are taken from the literature to be 5 Å^{-1} and 1.8 respectively[8].

Supplementary References

1. Best, R. B. *et al.* Optimization of the additive CHARMM all-atom protein force field targeting improved sampling of the backbone , and side-chain (1) and (2) dihedral angles. *J Chem Theory Comput* **8**, 3257–3273 (2012).

2. Jorgensen, W., Chandrasekhar, J., Madura, J., Impey, R. & Klein, M. Comparison of simple potential functions for simulating liquid water. *J. Chem. Phys.* **79**, 926 (1983).

3. McClendon, C., Friedland, G., Mobley, D., Amirkhani, H. & Jacobson, M. Quantifying correlations between allosteric sites in thermodynamic ensembles. *J Chem Theory Comput* **5**, 2486–2502 (2009).

4. Bowman, G. R. & Geissler, P. L. Extensive conformational heterogeneity within protein cores. *J Phys Chem B* **118**, 6417–6423 (2014).

5. Malmendal, A., Evenäs, J., Forsén, S. & Akke, M. Structural dynamics in the c-terminal domain of calmodulin at low calcium levels. *J. Mol. Bio.* **293**, 883–899 (1999).

6. Marlow, M. S., Dogan, J., Frederick, K. K., Valentine, K. G. & Wand, A. J. The role of conformational entropy in molecular recognition by calmodulin. *Nat. Chem. Biol.* **6**, 352–358 (2010).

7. Plummer, B. N. C. K., M. & Vines, K. CODA: Convergence diagnosis and output analysis for MCMC. *R News* **6** (2006).

8. Best, R. B., Hummer, G. & Eaton, W. A. Native contacts determine protein folding mechanisms in atomistic simulations. *Proc. Natl. Acad. Sci. U.S.A.* **110**, 17874–17879 (2013).
